# An auxin controls bacterial antibiotics production

**DOI:** 10.1093/nar/gky766

**Published:** 2018-08-24

**Authors:** Miguel A Matilla, Abdelali Daddaoua, Andrea Chini, Bertrand Morel, Tino Krell

**Affiliations:** 1 Department of Environmental Protection, Estación Experimental del Zaidín, Consejo Superior de Investigaciones Científicas, 18008 Granada, Spain; 2 Department of Plant Molecular Genetics, National Centre for Biotechnology, Consejo Superior de Investigaciones Científicas, 28049 Madrid, Spain; 3 Departament of Physical Chemistry and Institute for Biotechnology, Science Faculty, Granada University, 18071 Granada, Spain

## Abstract

The majority of clinically used antibiotics originate from bacteria. As the need for new antibiotics grows, large-scale genome sequencing and mining approaches are being used to identify novel antibiotics. However, this task is hampered by the fact that many antibiotic biosynthetic clusters are not expressed under laboratory conditions. One strategy to overcome this limitation is the identification of signals that activate the expression of silent biosynthetic pathways. Here, we report the use of high-throughput screening to identify signals that control the biosynthesis of the acetyl-CoA carboxylase inhibitor antibiotic andrimid in the broad-range antibiotic-producing rhizobacterium *Serratia plymuthica* A153. We reveal that the pathway-specific transcriptional activator AdmX recognizes the auxin indole-3-acetic acid (IAA). IAA binding causes conformational changes in AdmX that result in the inhibition of the expression of the andrimid cluster and the suppression of antibiotic production. We also show that IAA synthesis by pathogenic and beneficial plant-associated bacteria inhibits andrimid production in A153. Because IAA is a signalling molecule that is present across all domains of life, this study highlights the importance of intra- and inter-kingdom signalling in the regulation of antibiotic synthesis. Our discovery unravels, for the first time, an IAA-dependent molecular mechanism for the regulation of antibiotic synthesis.

## INTRODUCTION

Microbes represent a valuable source of antibiotics and around two-thirds of all naturally-derived antibiotics in clinical use are produced by bacteria (1). The ecological functions of these bioactive natural products are diverse and play important roles in inter-microbial and host-microbe interactions. Most frequently, antibiotics act as chemical warfare agents in the killing or inhibition of microbial competitors (2–5) and are used for either predation or protection purposes (3,6). In addition, bacterially produced antibiotics can serve to protect hosts from infections (1,2,7) and increasing evidence indicates that they may function at sub-inhibitory concentrations as inter- and intra-species signalling molecules that modulate gene expression and various cellular processes (3,8–10).

The genome of a bacterium can contain up to fifty gene clusters involved in antibiotic synthesis (1) and some strains devote up to 10% of their genomes to secondary metabolism (11,12). Because high metabolic costs are associated with the synthesis of these metabolites, their production is tightly regulated (3,5,9,13–15). This is reflected in the fact that many antibiotic biosynthetic clusters are cryptic and are not expressed under standard growth conditions (1,10,13,15) without the necessary environmental and physiological signals (5,9,10,13–15). The sensing of these signals is mainly achieved through global and pathway-specific transcriptional regulators that modulate the expression of antibiotic gene clusters in response to endogenous and exogenous cues. However, the signals recognized by most of the regulators involved in antibiotic production as well as their corresponding mechanisms of action remain largely unknown, which in turn hampers the discovery of novel antibiotics.

We have addressed this issue using the biocontrol root-associated bacterium *Serratia plymuthica* A153 as a model. The strain A153 produces a wide repertoire of antibiotics (16), including the halogenated antifungals oocydin A (17,18) and pyrrolnitrin (19), the polyamino antibiotic zeamine (20) and the broad spectrum antibacterial andrimid (21). This last antibiotic is a hybrid polyketide-nonribosomal peptide that is a highly efficient inhibitor of the bacterial acetyl-CoA carboxylase - an enzyme responsible for the first committed step of fatty acid synthesis (22). Different transcriptional and post-transcriptional regulators were found to modulate the expression of the andrimid (*adm*) biosynthetic cluster (21). One of them, the pathway-specific AdmX, was shown to activate the transcription of the *adm* gene cluster. Genome mining approaches revealed that this regulator is restricted to plant-associated bacteria (21), indicating that it may respond to niche-specific signals. Sequence analysis by Pfam (23) indicates that AdmX is composed of an N-terminal DNA-binding domain and a LysR-type ligand binding domain (LBD) at its C-terminal extension (Supplementary Figure S1). Based on the molecular mechanism of this family of transcriptional regulators (24), we hypothesized that the function of AdmX is controlled by the binding of specific signal molecules to its LBD.

Here, we used high-throughput screening (HTS) to identify signals that are recognized by AdmX. We then studied the function of these signals using a variety of *in vivo* and *in vitro* approaches. Our results highlight the significance of inter- and intra-kingdom signalling in the activation of antibiotic biosynthetic clusters.

## MATERIALS AND METHODS

### Reagents, primers, plasmids, strains and culture conditions

Indole-3-acetic acid (IAA) and indole-3-pyruvic acid (IPA) were purchased from Sigma-Aldrich (98% minimal purity). Bacterial strains are listed in Supplementary Table S1, whereas plasmids and primers are listed in Supplementary Tables S2 and S3, respectively. *Serratia* strains were routinely grown at 25°C, unless otherwise indicated, in either Luria-Bertani (LB) broth or minimal medium (0.1% (w/v) (NH_4_)_2_SO_4_, 0.41 mM MgSO_4_, 40 mM K_2_HPO_4_, 14.7 mM KH_2_PO_4_, pH 7.0) supplemented with 15 mM glucose.

### Protein overexpression and purification

The DNA fragment encoding AdmX (GenBank: KYQ97099) and its ligand binding domain (amino acids 65–295) were amplified by PCR and subsequently cloned into pET-based expression vectors (Novagen) to generate plasmids pMAMV232 and pMAMV235, respectively. These plasmids were transformed into *Escherichia coli* BL21-AI™ (Invitrogen) and cultures were grown at 30°C in LB medium. Protein expression was induced at an OD_660_ of 0.5 by the addition of 0.2% (w/v) l-arabinose and 0.5 mM isopropyl-β-d-thiogalactopyranoside (IPTG). Growth was then continued at 18°C overnight and cells were harvested by centrifugation at 6000 × *g* for 20 min. Proteins were purified by metal affinity chromatography using standard procedures and dialyzed into different buffer systems. Differential scanning fluorimetry (DSF) and isothermal titration calorimetry (ITC) experiments of AdmX and AdmX-LBD were conducted in 50 mM KH_2_PO_4_/K_2_HPO_4_, 300 mM NaCl, 10% (v/v) glycerol, 2 mM dithiothreitol, pH 7.0 and 20 mM HEPES, 150 M NaCl, 2 mM DTT, pH 7.4, respectively. Dynamic light scattering (DLS), attenuated total reflectance Fourier-transform infrared spectroscopy (ATR-FTIR) and circular dichroism spectroscopy (CD) measurements were performed in 50 mM KH_2_PO_4_/K_2_HPO_4_, 300 mM NaCl, 10% (v/v) glycerol, 2.5 mM β-mercaptoethanol, pH 7.0.

### Differential scanning fluorimetry (DSF)

Thermal shift assays were performed using a Bio-Rad MyIQ2 Real-Time PCR instrument. Assay mixtures (25 μl) contained 20 μM protein, SYPRO^®^ Orange (Life Technologies) at 5× concentration and ligands at final concentrations of 0.5–2 mM. Samples were heated from 23 to 85°C at a rate of 1°C min^−1^. The protein unfolding curves were monitored by detecting changes in SYPRO^®^ Orange fluorescence.

### Isothermal titration calorimetry (ITC)

Measurements were made using a VP-ITC titration calorimeter (Microcal Inc., Northampton, MA, USA) at 30°C for AdmX-LBD and 10°C (IAA) or 30°C (IPA) for AdmX. Proteins at 20–55 μM were titrated with 0.5–2 mM ligand solutions made in dialysis buffer. The mean enthalpies measured from the injection of ligands into the buffer were subtracted from raw data prior to data fitting using the ‘One binding site model’ of the MicroCal version of the ORIGIN software. In the absence of binding, experiments were repeated at a different analysis temperature.

### Dynamic light scattering (DLS)

Measurements were performed at 10°C on a Zetasizer μV dynamic light scattering instrument (Malvern Instruments, Worchestershire, UK). Zetasizer software (Malvern Instruments, Worchestershire, UK) was used for data collection and processing of the correlation function to obtain the particle size distributions. Shown are means of three measurements, each representing 20 scans with a duration of 10 s.

### Attenuated total reflectance-fourier transform infrared spectroscopy (ATR-FTIR)

Spectra were recorded at 10°C from 900 to 4000 cm^−1^ on a Bruker IFS-66 FTIR spectrometer (Bruker, Ettlingen, Germany) equipped with a liquid N_2_-cooled MCT detector and a BioATR-II cell. For each sample, 128 interferograms were recorded and Fourier transformed with a zero filling factor of 4 to yield spectra with a nominal resolution of 2 cm^−1^. Buffer spectra were recorded under identical conditions and subtracted from the spectra of the protein sample. Spectral contributions from residual water vapor were reduced using the atmospheric compensation filter of the Bruker OPUS software (Bruker, Ettlingen, Germany). The amide I band shape was fitted with a sum of Gaussian peaks using Origin 8.5 (OriginLab, Norhtampton, MA) to calculate the secondary structure content. The positions of the amide I band components were identified using the minima obtained from the second derivative of the spectra.

### Near-UV circular dichroism spectroscopy (CD)

Experiments were performed on a Jasco J-715 (Tokyo, Japan) spectropolarimeter equipped with a thermostatted cell holder. Measurements were made with a 5 mm path length quartz cuvette at a final protein concentration of 20 μM. Protein spectra were corrected with those of the ligands. Spectra shown are the means of 20 scans.

### Limited proteolysis

AdmX (10 μM) was incubated with 0.02 mg/ml trypsin (Sigma-Aldrich, catalog no. T8003) and 0.02 mg/ml α-chymotrypsin (Sigma-Aldrich, catalog no. C31421) in the absence or presence of 1 mM IAA or IPA at 25°C for 120 min. Samples were taken at regular intervals and reactions stopped by the addition of 5 μl 4× SDS sample buffer and subsequently analysed by electrophoresis on 15% (w/v) SDS*-*PAGE gels.

### Antibacterial and anti-oomycete assays

Antibacterial assays were carried out as previously described (20,21). For the assessment of andrimid production in the presence of bacterial supernatants, strains were grown at 30°C in minimal medium in the presence or absence of 1 mg/ml l-tryptophan (l-Trp). After 48 h, samples were taken, bacterial cells pelleted by centrifugation (10 000 × g, 5 min) and the supernatants filter-sterilized. Subsequently, A153 was grown at 25°C in minimal medium supplemented with supernatants from different IAA producing strains. After 24 h, the bioactivity of the supernatants was determined as previously described (21). Activities against the fast growing plant pathogenic oomycete *Pythium ultimum* were assayed as described previously (18).

### In-frame deletion mutagenesis

Chromosomal mutants of *S. plymuthica* strains were constructed by homologous recombination using derivative plasmids of the suicide vector pKNG101. These plasmids carried mutant in-frame deletions for the replacement of wild type genes and were transferred to *S. plymuthica* strains by triparental conjugation using *E. coli* CC118λ*pir* and *E. coli* HH26 (pNJ500) as helper. The in-frame deletion mutant strains RS02730 and RS14020 were generated using plasmids pMAMV267 and pMAMV268, respectively. Sucrose (10%, w/v) was used to select derivatives that had undergone a second crossover event. When required, the generalized transducing bacteriophage ϕMAM1 was used for transduction of chromosomal mutations, as previously described (25).

### β-Galactosidase assays

Assays were carried out in *S. plymuthica* A153 LacA (control) or derived mutants following the previously reported protocol (26).

### Electrophoretic mobility shift assays (EMSA)

Promoter fragments were amplified by PCR and end-labelled with [γ-^32^P] ATP (Perkin Elmer) using T4 polynucleotide kinase (Roche). Unincorporated [γ-^32^P] ATP was removed by Bio-Gel P-6 polyacrylamide gel spin columns (Bio-Rad). Standard ten microliter samples in binding buffer (50 mM Tris/HCl, 100 mM KCl, 10 mM MgCl_2_, 10% (v/v) glycerol, 0.025 mM EDTA, 0.4 mM DTT, pH 8.5) contained 2 nM of labeled DNA (1.5 × 10^4^ c.p.m.), different AdmX concentrations, 50 μg ml^−1^ poly[d(I-C)] (Roche) and 1 mg ml^−1^ bovine serum albumin (BSA). These samples were incubated at 30°C for 20 min to allow complex formation. DNA–protein complexes were resolved in non-denaturing 4% (w/v) polyacrylamide gels run in Tris-glycine buffer. Gels were scanned on a phosphoimager.

### Primer extension analysis

Total RNA was isolated from cultures grown in LB to an OD_600_ of 4.0 using TRI Reagent (Ambion, Austin, USA), followed by DNase treatment. Primer extension analyses were carried out using 30 μg of total RNA following the protocol described previously (27).

### *In vitro* transcription assays

*In vitro* transcription reactions (20 μl) from P*_adm_* were performed in 50 mM Tris–HCl, 100 mM KCl, 10 mM MgCl_2_, 10% (v/v) glycerol, 0.025 mM EDTA, 0.4 mM DTT, pH 8.5, containing 0.5 units of σ^70^*-*saturated *E. coli* RNA polymerase holoenzyme (New England Biolabs, catalog no. M0551S), 300 ng circular P*_adm_* DNA template (pMAMV286), 10 μM AdmX and different concentrations of IAA and IPA. Mixtures were incubated at 30°C for 10 min prior to the addition of ATP, CTP, GTP (final concentration of 0.1 mM), UTP (final concentration of 0.05 mM) and 3.6 μCi of [α-^32^P]UTP (10 μCi/ml). After incubation for 1 h at 30°C, the reactions were stopped by incubating at 95°C for 10 min, then chilled to 4°C at which point 4 μl of formamide sequencing dye was added. *In vitro* transcription assays from the P*_gap-1_*promoter *Pseudomonas aeruginosa* PAO1 were performed as previously described (28). All samples were separated on 6.5% (w/v) polyacrylamide gels. Gels were scanned on a phosphorimager and densitometric analyses were carried out using Quantity One Analysis software v.4.6.1 (Bio-Rad Laboratories) with final values representing the background-subtracted density of the bands. For the generation images, brightness and contrast have been adjusted uniformly using the Quantity One analysis software.

### Quantification of IAA in bacterial supernatants

The colorimetric Salkowski assay was used (29). Strains were grown in either LB or minimal media supplemented with different concentrations of l-Trp. After 48 h at 30°C, 1 ml samples were taken and cells centrifuged (13 000 × *g*, 5 min). The resulting supernatants were mixed with 2 ml of Salkowski's reagent and incubated at room temperature for 30 min before measuring at OD_535_. IAA concentrations were inferred from a standard curve obtained with commercial IAA (Sigma-Aldrich).

### *In vitro* plant growth and sampling

*Arabidopsis thaliana* Col-0 is the genetic background of wild type and mutant lines used in this study (Supplementary Table S1). Seeds were surface-sterilized by the chlorine gas method and stratified for 2–3 days at 4°C in the dark. Seedlings were grown vertically on Murashige and Skoog medium plates under a 16-h light/8-h dark cycle at 21°C. Ten-day-old seedlings and roots from 10-day-old plants (∼100 plants) were collected and frozen. Alternatively, wild type seedlings were treated with 20 μM IAA for 24 h. Root extract was obtained by grinding roots in liquid nitrogen with a mortar and pestle.

## RESULTS

### Identification of indole-3-acetic and indole-3-pyruvic acids as AdmX ligands

In order to identify AdmX signals, we produced recombinant AdmX-LBD for use in a HTS assay. The assay that we developed provides a readout of the thermal stability of the protein, or more specifically, the melting temperature (*T*_m_)—a value that corresponds to the midpoint of the protein unfolding transition (30). Typically, ligand binding stabilizes the protein and Tm increases by 2°C or more are considered significant (30). Thus, we screened a collection of ∼1700 ligands that included (a) andrimid synthesis precursors; (b) 450 compounds that serve as bacterial carbon, nitrogen, phosphorous or sulphur sources; (c) approximately 1,200 compounds of a Natural Product-Like Library, which includes an array of natural product-like scaffolds (31) and (d) a collection of 43 natural and synthetic auxins (Supplementary Table S4).

**Figure 1.**
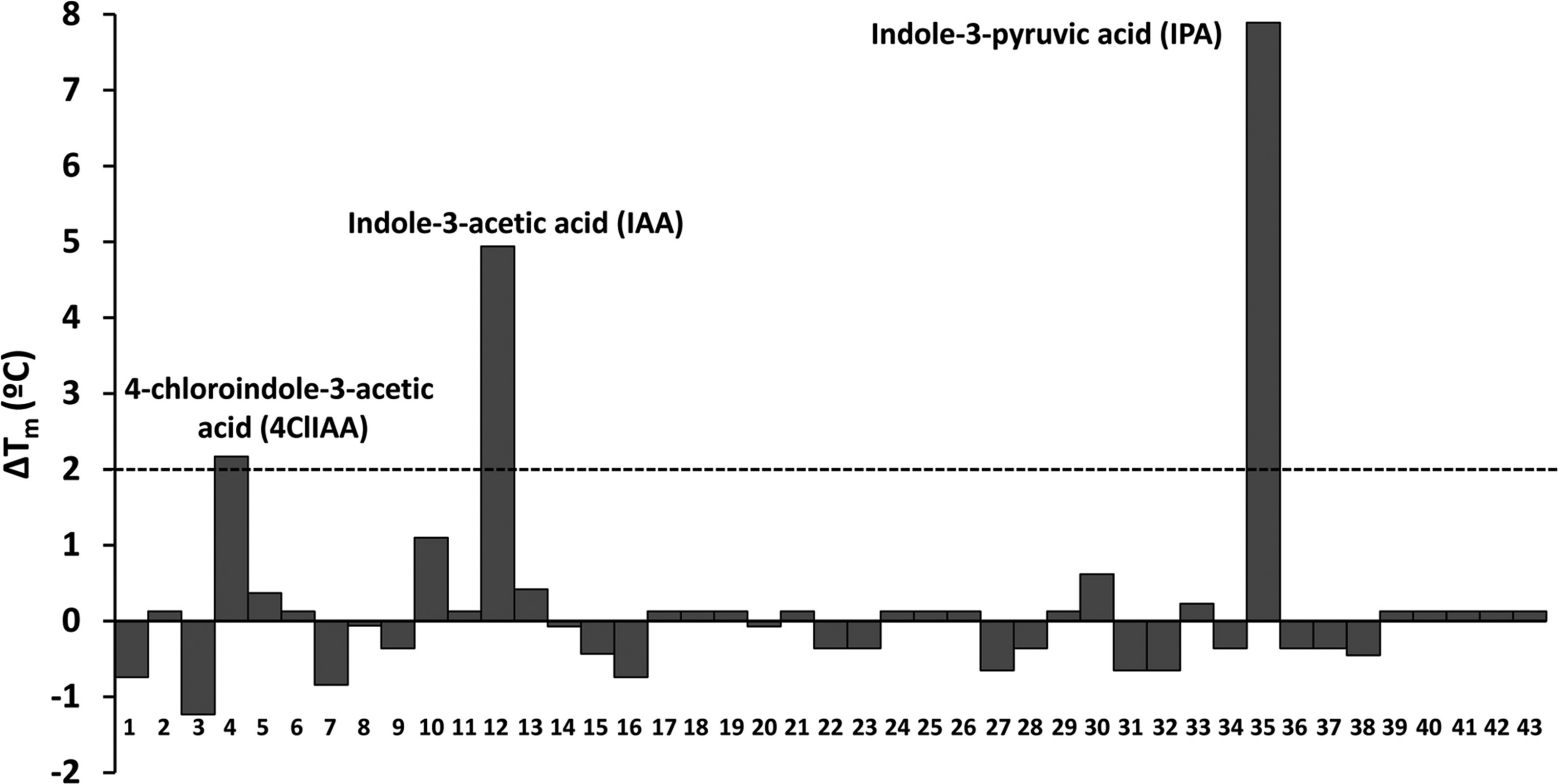
High-throughput screen to identify ligands recognized by the AdmX ligand binding domain. Shown are changes in the melting temperature (*T*_m_) caused by the presence of different auxins (Supplementary Table S4). Compounds that caused *T*_m_ shifts of 2°C or greater are annotated.

Ligand free AdmX-LBD showed a *T*_m_ of 56.5°C and our screen detected three compounds that caused significant increases in *T*_m_, namely 4-chloroindole-3-acetic acid (4ClIAA), indole-3-acetic acid (IAA) and indole-3-pyruvic acid (IPA) (Figure 1). 4ClIAA and IAA are naturally occurring auxins, while IPA is an auxin biosynthetic intermediate. To confirm binding, we conducted isothermal titration calorimetry (ITC) studies with purified AdmX-LBD and found binding for IAA and IPA with dissociation constants (*K*_D_) of 15.2 and 6.4 μM, respectively (Supplementary Figure S2). However, no binding between purified AdmX-LBD and 4ClIAA was observed, which is likely due to the fact that ITC permits only the detection of high-affinity binding events.

**Figure 2.**
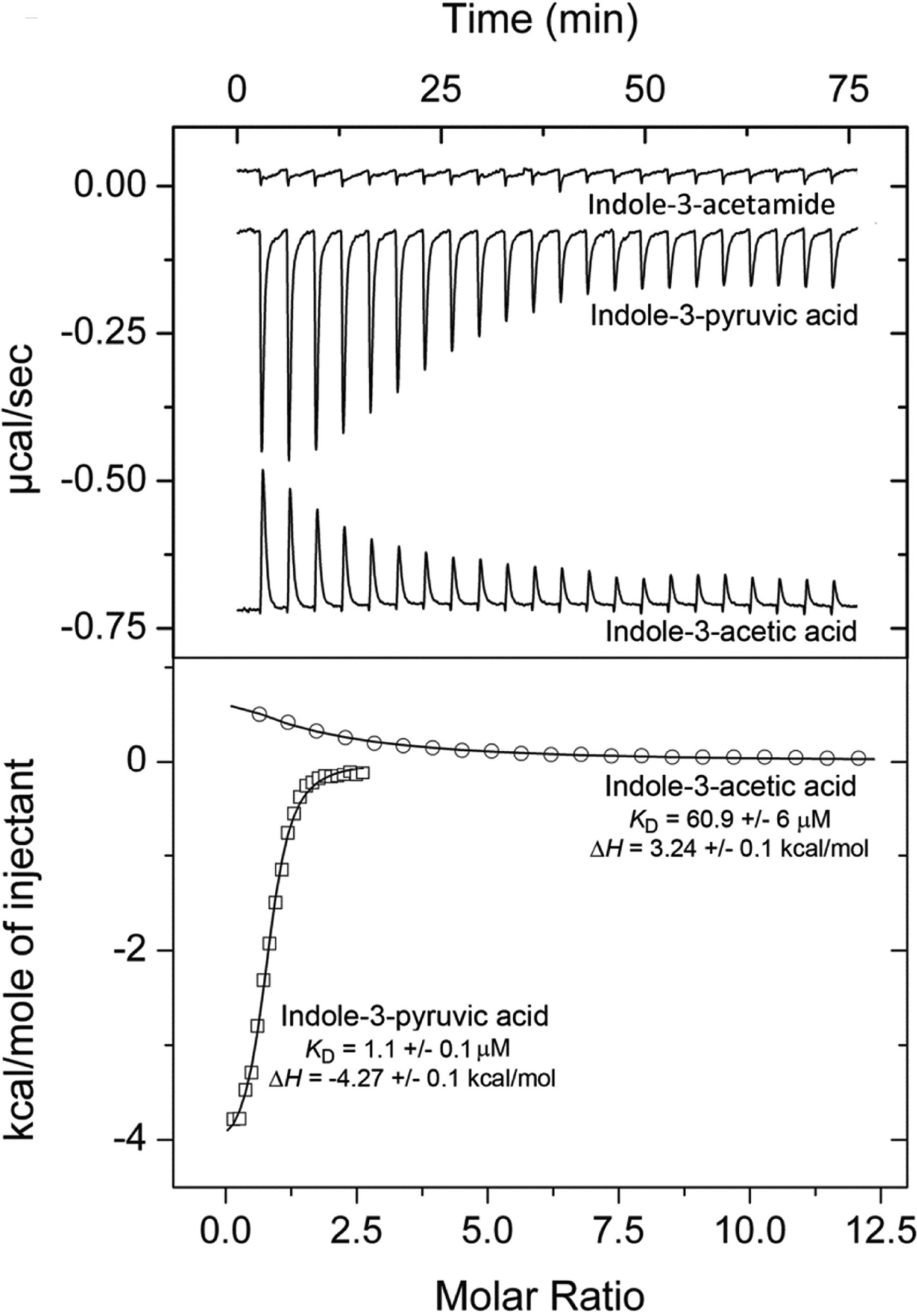
Isothermal titration calorimetry showing the binding of auxinic compounds to AdmX. Upper panel: Raw data for the titration of 21 μM AdmX with indole-3-acetic acid (IAA; 2 mM), indole-3-pyruvic acid (IPA; 0.5 mM) and a saturated indole-3-acetamide solution (IAM). Lower panel: Integrated, dilution heat-corrected and concentration-normalized peak areas fitted using ‘One binding site’ model of ORIGIN software. The assays were repeated at least three times and a representative figure is shown.

We then generated full-length AdmX and studied IAA and IPA binding. In accordance with our findings using the AdmX-LBD, IAA and IPA caused Tm increases of 6.0 and 9.6°C, respectively, in the full-length protein (Supplementary Figure S3). Titration of AdmX with IAA or IPA resulted in *K*_D_ of 60.9 μM and 1.1 μM, respectively (Figure 2). Interestingly, IAA binding led to unfavourable enthalpy changes (upwards going peaks), whereas IPA binding was characterized by favourable enthalpy changes (downwards going peaks). Because these two ligands have very similar structures, we hypothesize that the observed enthalpy change differences may be caused by differential structural alterations that are induced upon binding.

After verifying that AdmX recognizes two auxinic compounds with significant affinity, we carried out subsequent ITC experiments to explore whether related compounds were also part of the ligand profile. In bacteria, IAA is mainly synthesized from l-tryptophan (l-Trp) via five biosynthetic pathways (Supplementary Figure S4) (32). However, microcalorimetric titrations with intermediates of the different biosynthetic pathways (Supplementary Figure S4) did not reveal any binding, as exemplified by indole-3-acetamide in Figure 2. Subsequently, microcalorimetric titrations with additional related compounds, including the natural auxins indole-3-butyric acid and 2-phenylacetic acid as well as the auxinic compounds 5-hydroxyindole-3-acetic and indole-3-carboxylic acids, also failed to show binding. Therefore, we conclude that AdmX specifically recognizes the natural auxin IAA as well as IPA, an intermediate of the main IAA biosynthetic pathway in plants and plant beneficial bacteria (32,33).

### Suppression of andrimid production by Indole-3-acetic and indole-3-pyruvic acids

**Figure 3.**
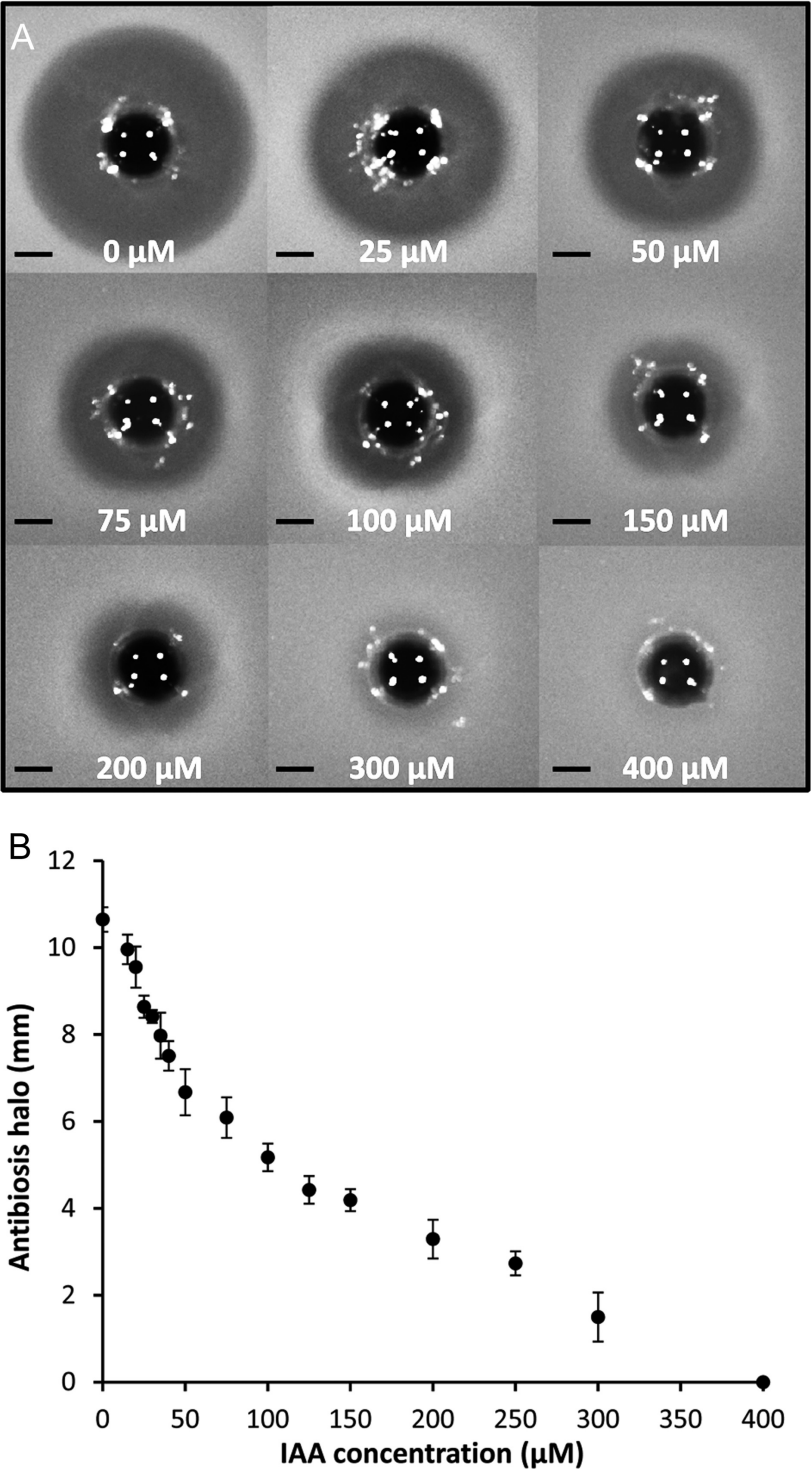
Indole-3-acetic acid inhibits andrimid biosynthesis in *Serratia plymuthica* A153. (**A**) Andrimid production by *S. plymuthica* A153 strain JH6 (zeamine negative) grown in minimal medium in the presence of increasing IAA concentrations. For the assays, a *Bacillus subtilis* agar lawn was prepared and 400 μl of filter-sterilized supernatants were added to holes punched into the plates. Bars, 5 mm. (**B**) Quantification of andrimid-induced inhibition halos derived from the assays shown in (A). Means and standard deviations from three individual experiments are shown.

To evaluate whether IAA and IPA control andrimid biosynthesis, we used a zeamine mutant of A153 and therefore defective in the only other antibacterial compound produced in this strain. The mutant was grown in minimal medium supplemented with IAA or IPA across a range of concentrations that do not affect the growth of A153 (i.e. 0–2 mM) (Supplementary Figure S5). At different times, the presence of andrimid in filter-sterilized supernatants was measured by monitoring their antibacterial activity against *Bacillus subtilis*. The assays revealed that the exogenous addition of IAA suppressed the andrimid-mediated antibacterial properties of A153 (Figure 3). The inhibitory effect was observed at a concentration as low as 10 μM IAA and the production of the antibiotic was totally abolished at 400 μM of the auxin (Figure 3). Dose-response measurements resulted in an EC_50_ of 96 ± 5 μM (Figure 3B), which is only slightly higher than the *K*_D_ value (i.e. 60.9 μM) reported above.

Our results also showed that IPA prevented andrimid synthesis, but to a much lesser extent. The initial suppression of antibiotic activity was observed at 500 μM IPA, while all antibiotic activity was abolished at a concentration of 2 mM IPA (Supplementary Figure S6). In order to determine whether IAA plays a specific role in andrimid biosynthesis, we analysed its effect on the production of other bioactive secondary metabolites in A153, including the antifungal and anti-oomycete oocydin A (17,18) as well as the antibacterial zeamine (20). The bioassays showed that neither zeamine nor oocydin A production were affected by the addition of IAA (Supplementary Figure S7A and B). However, we observed an inhibition of zeamine synthesis at 500 μM or greater IPA (Supplementary Figure S7C)—the same concentration at which IPA began to suppress andrimid production (Supplementary Figure S6).

### AdmX-mediated transcription is inhibited by indole-3-acetic and indole-3-pyruvic acids

**Figure 4.**
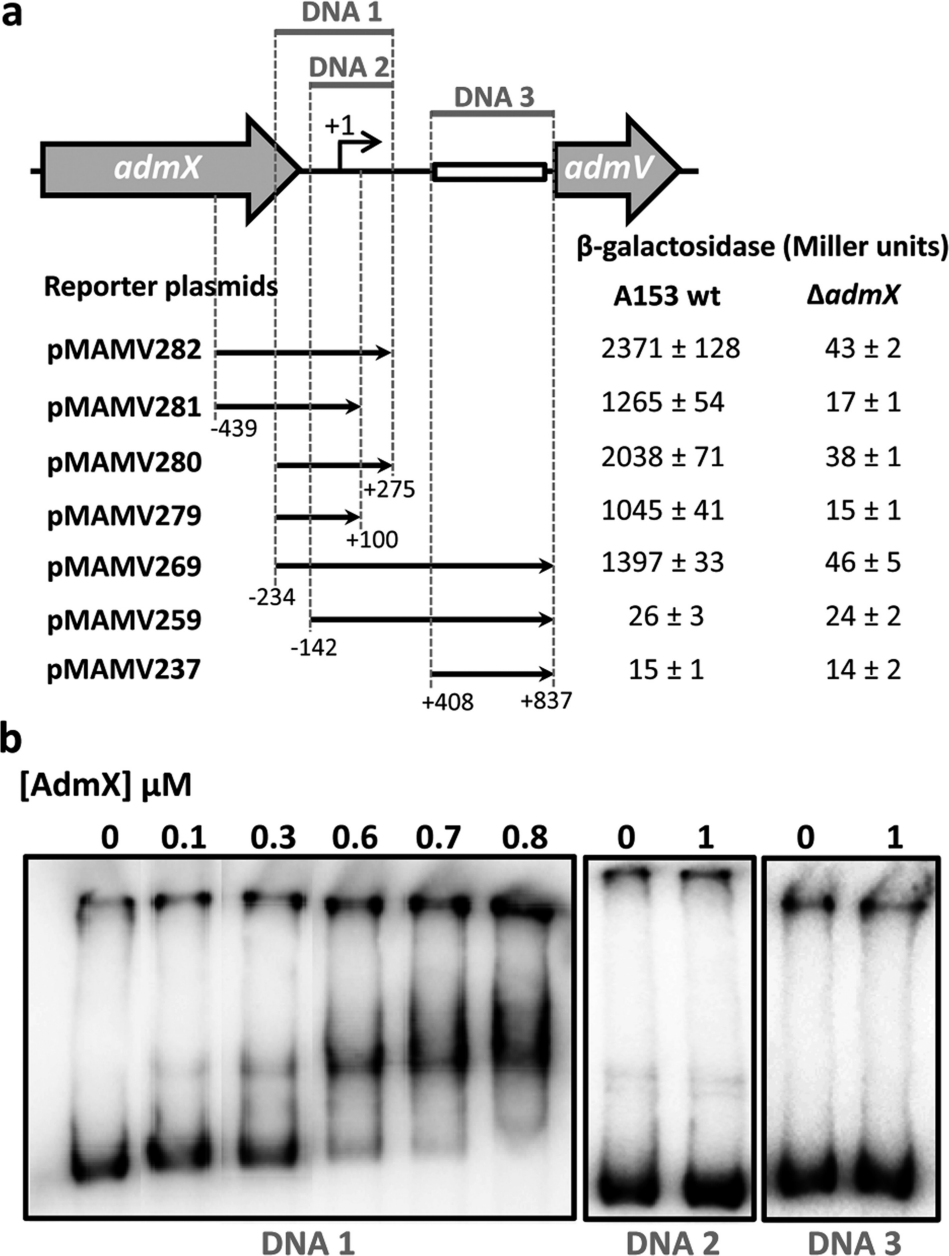
Characterization of the *adm* promoter of *Serratia plymuthica* A153. (**A**) Schematic representation of the promoter of the *adm* biosynthetic cluster and β-galactosidase assay results (after 10 h incubation at 25°C in LB medium) of *S. plymuthica* A153 harbouring different reporter plasmids. Black arrows indicate the DNA fragment that has been fused to the *lacZ* reporter gene. The box indicates the location of sequences with homology to transposable genetic elements. (**B**) Electrophoretic mobility shifts assays of different DNA fragments with AdmX. The size and genomic location of the DNA fragments tested are defined in (A).

We previously showed that the andrimid gene cluster consists of a single transcriptional unit of which *admV* is the first gene (21). To assess the effect of IAA and IPA on the transcription of the *adm* biosynthetic cluster, we characterized the promoter upstream of *admV* (P*_adm_*). Initial analysis revealed the presence of a ∼0.4 kb region showing homology to transposable genetic elements (Supplementary Figure S8a,b), which suggests that the *adm* gene cluster was acquired through horizontal gene transfer. Primer extension analyses were conducted and allowed the identification of a guanine located 834 bp upstream of the translation start codon of *admV* as the transcriptional start site (Supplementary Figure S8A, C). Next, to define the promoter region, we created transcriptional fusions containing different sequences between *admX* and *admV* within a β-galactosidase reporter plasmid (Figure 4A). We then measured β-galactosidase activities in wild type A153 and in an *admX* mutant strain. The assays showed that P*_adm_* overlaps with the 3′ end of *admX*. Also, these assays revealed that a ∼450 bp fragment immediately upstream from the *admV* ATG start codon (which includes remnants of transposable elements) is not required for transcription from P*adm* (Figure 4A). In agreement with these data, electrophoretic mobility shift assays revealed that AdmX specifically binds to a region within P*_adm_* that includes the 3′ end of *admX* (Figure 4B). Transcriptional regulators of the LysR-family recognize the consensus T-N_11_-A sequence (24) and eight putative LTTR boxes were identified within the region recognized by AdmX (Supplementary Figure S8A). To determine whether IAA alters the ability of AdmX to bind DNA, we carried out EMSA assays, but found no binding changes (Supplementary Figure S9). In light of this finding, and considering our ITC data (Figure 2), we hypothesized that IAA binding may cause conformational alterations that do not modulate promoter affinity.

**Figure 5.**
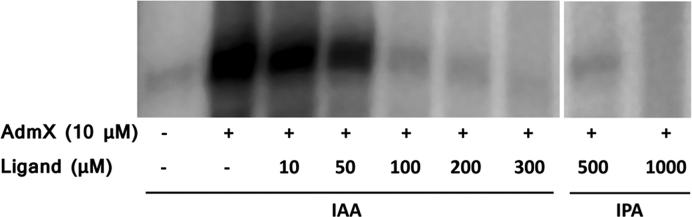
Determination of the effect of indole-3-acetic acid (IAA) and indole-3-pyruvic acid (IPA) on the capacity of AdmX to activate *in vitro* transcription of the andrimid biosynthetic cluster. Shown are *in vitro* transcription assays in the presence and absence of purified AdmX, and IAA and IPA at various concentrations. The assay was repeated at least three times and a representative gel is shown.

Subsequently, we carried out *in vitro* transcription assays, which confirmed that AdmX promotes transcription of the *adm* biosynthetic cluster and that IAA represses this transcription in a concentration-dependent manner (Figure 5). Thus, subtle repression occurred at 10 μM IAA, whereas at least 100 μM of the auxin was required for complete repression of transcription (Figure 5, Supplementary Figure S10). Control experiments showed that IAA did not significantly affect transcription from an AdmX independent promoter (Supplementary Figure S11), namely the P*_gap-1_* promoter of *Pseudomonas aeruginosa* (28). We also evaluated the effect of IPA and found that it was able to fully repress transcription from P*_adm_* at concentrations of 500 and 1000 μM (Figure 5).

The above mentioned data are consistent with the notion that IAA binding causes an alteration of AdmX that in turn modulates its effect on RNA polymerase activity. A series of experiments were conducted to verify the existence of such conformational changes. First, the hydrodynamic radius obtained in dynamic light scattering experiments indicated that AdmX is present in a higher order oligomeric state and that the addition of IAA and IPA resulted in an increase in the compactness of AdmX (Supplementary Figure S12A). Second, analyses by ATR-FTIR and near-UV circular dichroism spectroscopy revealed that IAA and IPA binding caused significant changes to the AdmX secondary structure (Supplementary Figure S12b,c; Supplementary Table S5). Furthermore, these data are consistent with limited proteolysis studies revealing structural changes upon ligand binding (Supplementary Figure S13).

Using β-galactosidase assays we then confirmed that IAA was able to modulate the expression of the *adm* gene cluster *in vivo* in A153. Repression occurred throughout growth and in a dose-dependent manner; however, higher concentrations of IAA were required compared to our *in vitro* transcription assays (Supplementary Figure S14).

### Reduction of endogenous indole-3-acetic acid synthesis has no effect on andrimid production

IAA in beneficial rhizobacteria is mainly synthesized from l-Trp through the indole-3-pyruvate pathway (Supplementary Figure S4) (32–34). To determine whether endogenously produced IAA modulates andrimid synthesis, we first investigated the capacity of A153 to produce IAA. Our results showed that in the absence of L-Trp, A153 produced none or very low IAA levels (Supplementary Figure S15). However, supplementation of LB media (but not minimal media) with tryptophan resulted in high IAA levels (Supplementary Figures S15 and S16). Genome analysis of *S. plymuthica* A153 (16) revealed the presence of two genes, *AWY96_RS02730* and *AWY96_14020*, encoding putative indole-3-pyruvate decarboxylases that may be involved in the decarboxylation of IPA to indole-3-acetaldehyde (Supplementary Figure S4). We mutated these genes and found that deletion of *AWY96_14020* caused a 90% reduction in IAA synthesis compared to wild type levels, whereas the mutation of *AWY96_RS02730* did not affect IAA production (Supplementary Figure S15). We then tested whether loss of *AWY96_14020* affected andrimid production in A153 when grown under conditions that promote high levels of IAA (i.e. rich medium supplemented with l-Trp). Under these conditions, loss of *AWY96_14020* had no effect on expression of the *adm* cluster and andrimid production (Supplementary Figure S17).

### Exogenously-produced indole-3-acetic acid modulates andrimid production

**Figure 6.**
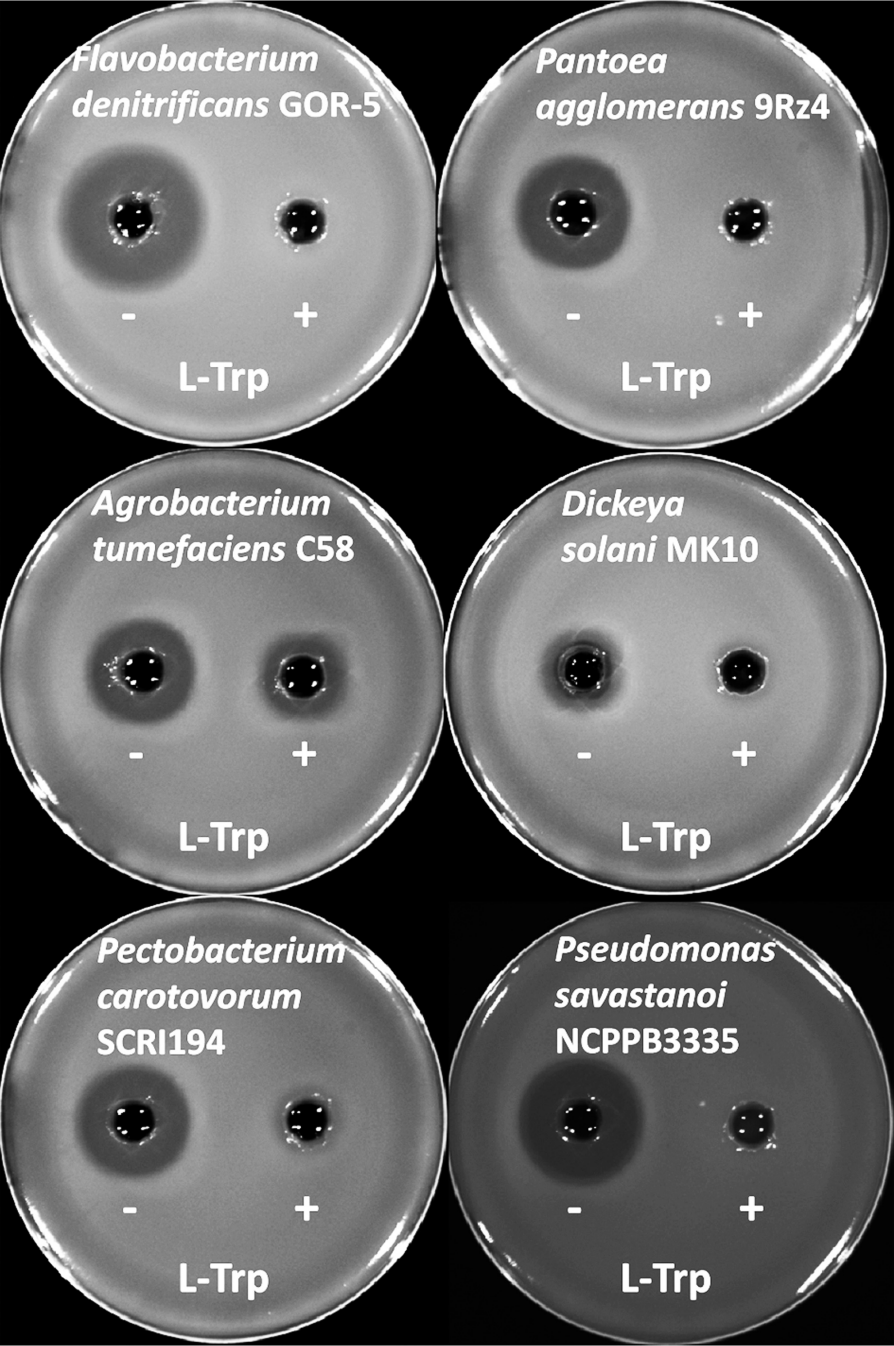
Indole-3-acetic acid (IAA) produced by various beneficial and pathogenic bacteria inhibits andrimid synthesis in *Serratia plymuthica* A153. Shown are antibiotic assays that indicate andrimid production by *S. plymuthica* A153 strain JH6 (zeamine negative) in minimal medium supplemented with supernatants from different bacterial cultures grown in the absence (−) and presence (+) of l-tryptophan.

To investigate the effect of IAA produced by other bacteria on the antagonistic properties of A153, several pathogenic and beneficial plant-associated bacteria were analysed for their ability to synthesize and secrete the auxin. These bacteria were grown in rich and minimal media and IAA concentrations in supernatants were measured in the presence and absence of l-Trp. In agreement with previous reports (32), most of the analysed plant-associated strains were able to synthesize IAA (Supplementary Figure S16). We found that levels of IAA varied depending on the presence of l-Trp in the culture media, and we also observed large differences in IAA concentrations between strains, which ranged from the lower micromolar to millimolar concentrations (Supplementary Figure S16). Next, we explored the effect of exogenously-produced IAA on andrimid synthesis in a zeamine-deficient mutant of A153. For these assays, we selected two beneficial and four pathogenic bacterial strains that produce high IAA levels in minimal medium supplemented with l-Trp, but greatly reduced IAA levels in the absence of the l-amino acid (Supplementary Figure S16b). Supernatants from these six strains grown in minimal medium in the presence or absence of l-Trp were prepared, filter-sterilized and then added to A153 cultures grown in minimal medium. The results revealed that andrimid production was either reduced or abolished by the supernatants of strains grown in the presence of l-Trp (Figure 6), indicating that IAA production by beneficial and phytopathogenic bacteria drastically inhibits andrimid synthesis. Control experiments in which 15 mM l-Trp was added to A153 cultures did not reveal any effect on bacterial growth or a reduction in andrimid production (Supplementary Figure S18).

We also evaluated whether plant-derived IAA modulates andrimid production. To this end, we carried out antibiotic assays using a zeamine negative derivative of A153 that was exposed to root extracts from (a) wild type *Arabidopsis thaliana*; (b) *A. thaliana* mutants that are known to produce either increased (*wei8/tar2, axr1-3*) or reduced (*sur2*) IAA levels; and (c) *A. thaliana* exogenously treated with IAA. The results showed that antibiotic activities were unchanged in all conditions (Supplementary Figure S19), which suggests that the effect of plant-produced IAA may be restricted to local areas with elevated IAA concentrations.

## DISCUSSION

The majority of the signals known to control antibiotic biosynthesis are derived from either the primary or secondary bacterial metabolism, including quorum sensing (QS) molecules (9,14,17), second messengers (13,35), nutritional and stress signals (3,9,13,14), or antibiotics and their biosynthetic intermediates (9,10,13,36). Additionally, numerous studies of bacterial community interactions have revealed that mostly unidentified intra/inter-species signals play an important role in regulating the expression of antibiotic gene clusters, including those involved in the production of cryptic antibiotics (3,4,9,13,15,37). The novelty of this study resides in the demonstration that antibiotic synthesis is controlled by an auxin. IAA is the main auxin produced by higher plants and a key plant hormone and regulator of plant growth and development (38). However, IAA has also been shown to be synthesized in archaea (39), fungi (40), animals (41) and in a wide array of bacteria (32,33) where it regulates the expression of genes involved in central metabolism (42–44), nitrogen fixation (44), adaptation to hosts (43,44) and stress (44,45). Here we identify a bacterial signalling system that controls antibiotic synthesis via a signal molecule that is omnipresent across all kingdoms of life. This finding reveals an important role for IAA in intra- and inter-kingdom signalling. In support of this notion, several studies have shown that microbially-produced IAA is involved in the suppression of plant defence responses, development of plant diseases, and plant growth promotion (32–34,46,47), as well as acting as a promoter of cell division in diatoms (48).

Our findings may also support the existence of a general mechanism through which bacteria regulate antibiotics in order to thrive in complex niches, especially because complementary studies exist that suggest that IAA can regulate the synthesis of other antibiotics (49,50). In this study, we established, for the first time, the molecular mechanism for the IAA-dependent regulation of antibiotic biosynthesis. Our results reveal that the direct recognition of IAA by a transcriptional regulator results in reduced transcription of the andrimid biosynthetic cluster and inhibition of antibiotic production. This regulatory mechanism may be widespread within bacteria and not only restricted to plant-associated strains of the *Serratia* genus. In fact, BLAST analyses revealed that uncharacterized AdmX homologs are present in enterobacteria belonging to the *Escherichia, Klebsiella, Pluralibacter* and *Raoultella* genera (Supplementary Figure S20).

The natural habitat of *S. plymuthica* A153 is the rhizosphere (16), a niche where IAA can mainly originate from two different sources: it can be released from plant roots (51,52) and it can be secreted by plant-associated bacteria (32,33). Indeed, some studies indicate that most of the characterized root-associated bacteria are able to produce IAA (32–34) and that its synthesis is frequently induced in the presence of root exudates and during the colonization of plant surfaces (32,34). Although the presence of IAA in root exudates has been shown to be in the nanomolar range (52,53), it was estimated that root-associated bacteria can produce 10 μM IAA in the rhizosphere (54)—a concentration that inhibits andrimid biosynthesis (Figure 3). Taken together, our results suggest that rhizosphere colonization by A153 causes a reduction in andrimid biosynthesis. This view is also supported by reports showing that the biosynthesis of different antibiotics produced by root-associated bacteria can be modulated by the rhizosphere microenvironment or by the presence of root exudates when compared to standard *in vitro* culture conditions (5,12,55,56). In accordance with this, the effect of exogenously provided IAA on andrimid production by A153 was found to depend on the culture conditions. Whereas in minimal medium 400 μM IAA are sufficient to suppress andrimid activity (Figure 3), ∼7.5 mM IAA are required when cells are grown in rich LB medium (Supplementary Figure S18b). We hypothesize that LB medium contains compounds with antagonistic action and future studies are necessary to decipher the molecular mechanisms behind this growth medium dependence.

Besides the above mentioned effects of IAA on plant-bacteria interactions, this work emphasizes that IAA behaves as a potent inter-species signalling molecule by interfering with antibiotic synthesis. Interestingly, several biological control agents and phytopathogenic bacteria are sensitive to andrimid (21,57). Here, we showed that the production of IAA by some of these andrimid-sensitive strains inhibits the synthesis of the antibiotic (Figure 6). This inhibition is likely to benefit IAA producers in a competitive social context by providing a dual advantage. First, it may favour their survival in particularly competitive nutrient-limited environments (i.e. soils) and could also aid in providing access to certain nutrient-rich ecological niches (i.e. rhizosphere). Second, it may also provide certain plant pathogens with an advantage against bacterial antagonists, ultimately enhancing their ability to infect plants.

The capability of IAA to modulate bacterial gene expression (32–34), together with its membrane permeability (34), suggests that it can act as a QS signalling molecule (32). Our previous work showed that an *N*-acyl-l-homoserine lactone-based QS system does not control andrimid production in A153 (21). However, A153 may rely on endogenously synthesized IAA to regulate antibiotic synthesis in a manner dependent on cell density under specific environmental and nutritional conditions. Future work is needed to further explore the potential role of IAA as a key signalling molecule within complex and competitive niches.

## Supplementary Material

Supplementary Data
