# Supplementary material for: An auxin controls bacterial antibiotics production
Source: Nucleic Acids Res. 2018 Aug 24;46(21):11229–38. doi: 10.1093/nar/gky766 (PMC6265452; doi:10.1093/nar/gky766)
Supplement: Supplementary Data [file gky766_supplemental_files.docx]

**Supplementary data**

**to**

**An auxin controls bacterial antibiotics production**

by

Miguel A. Matilla, Abdelali Daddaoua, Andrea Chini, Bertrand Morel and Tino Krell

**
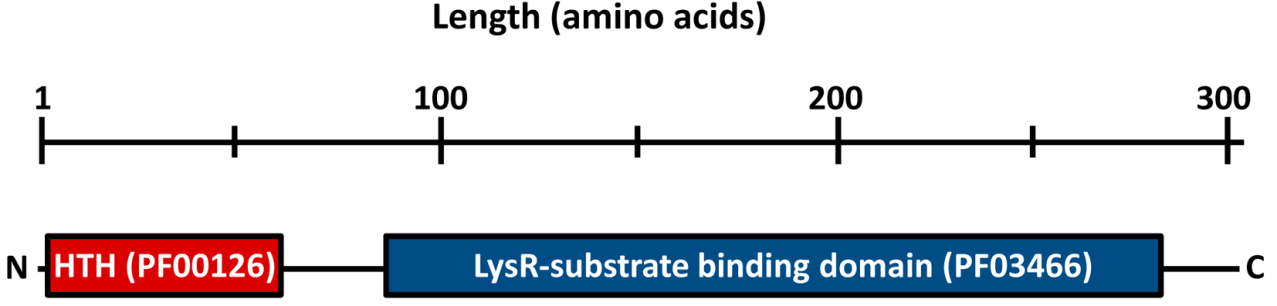
**

**Fig. S1) Domain organization of AdmX according to the Pfam (1) domain family database.** Pfam accession numbers of the corresponding domains are also shown. HTH, helix-turn-helix DNA binding domain.

**

**

**Fig. S2) Isothermal titration calorimetry studies for the binding of indole-3-acetic and indole-3-pyruvic acids to the AdmX ligand binding domain (LBD).** Upper panel: Raw data for the titration of 55 µM of AdmX-LBD with 5-hydroxyindole-3-acetic acid (5OHIAA; 2 mM), indole-3-acetic acid (IAA, 2 mM) and indole-3-pyruvic acid (IPA; 1 mM). Lower panel: Integrated, dilution heat-corrected and concentration-normalized peak areas fitted using “One binding site” model of ORIGIN software. Derived thermodynamic data are shown. The assays were repeated at least three times and a representative figure is shown.


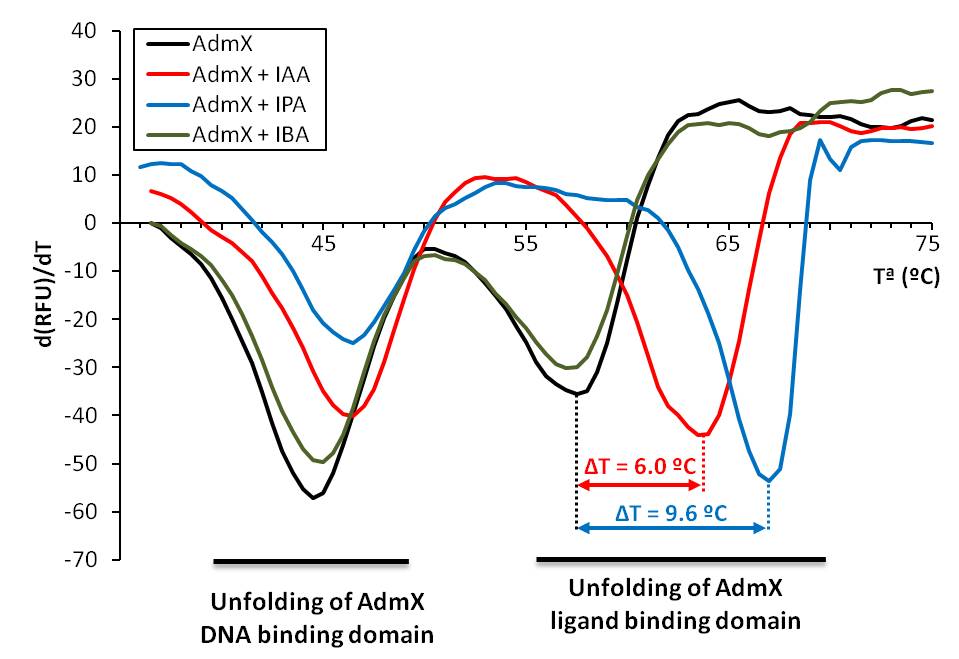


**Fig. S3) Thermal unfolding of full-length AdmX monitored by differential scanning fluorimetry.** Shown are thermograms in the presence and absence of 1 mM indole-3-acetic acid (IAA), indole-3-pyruvic acid (IPA) and indole-3-butyric acid (ABA; negative control). AdmX unfolds in two events: the initial event centered at around 45 °C represents unfolding of the DNA-binding domain, whereas the second event corresponds to the unfolding of the effector binding domain. The melting temperature (Tm) of this second event was increased by 6.0 and 9.6 °C for IAA and IPA, respectively, whereas the Tm in the presence of ABA remained unchanged.

**
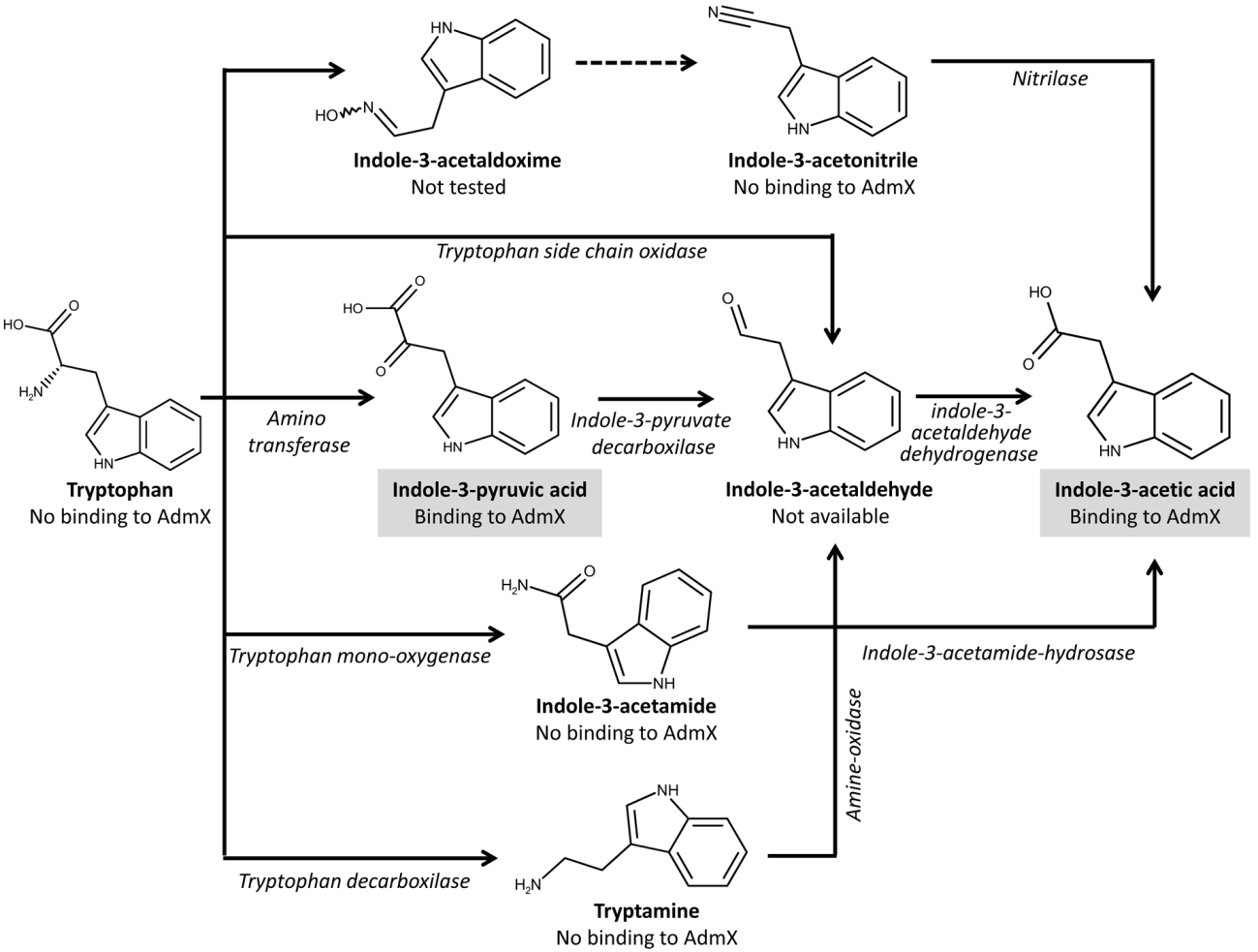
**

**Fig. S4) Indole-3-acetic acid biosynthesis pathways in bacteria.** This schematic was adapted from Spaepen & Vanderleyden (2). Binding of these compounds to AdmX in ITC experiments is indicated.


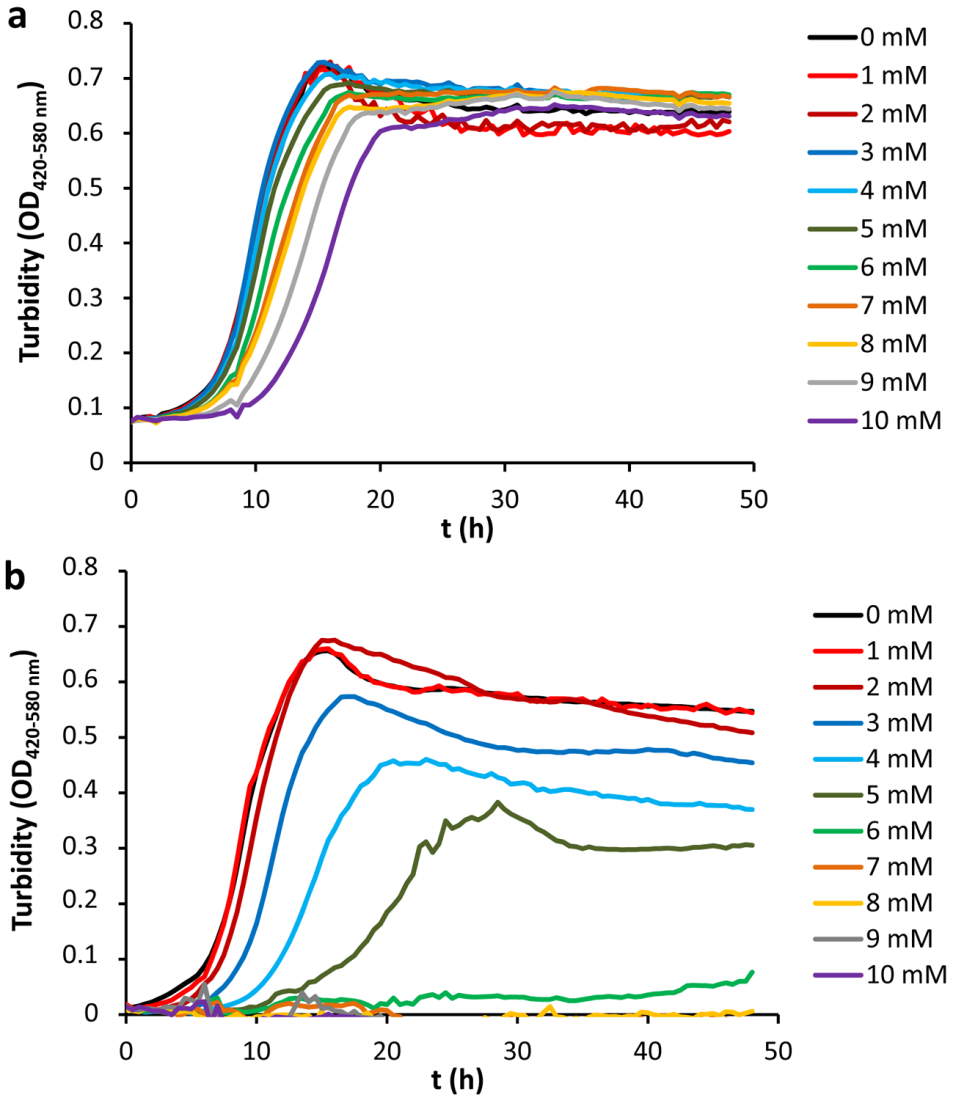


**Fig. S5) Growth curves of *Serratia plymuthica* A153 with different concentrations of IAA (a) and IPA (b).** Growth experiments were conducted in minimal medium with 15 mM glucose as carbon source at 30 ºC. Growth was measured using Bioscreen Microbiological Growth Analyser (Oy Growth Curves Ab Ltd, Helsinki, Finland). Data are means and from three biological replicates.


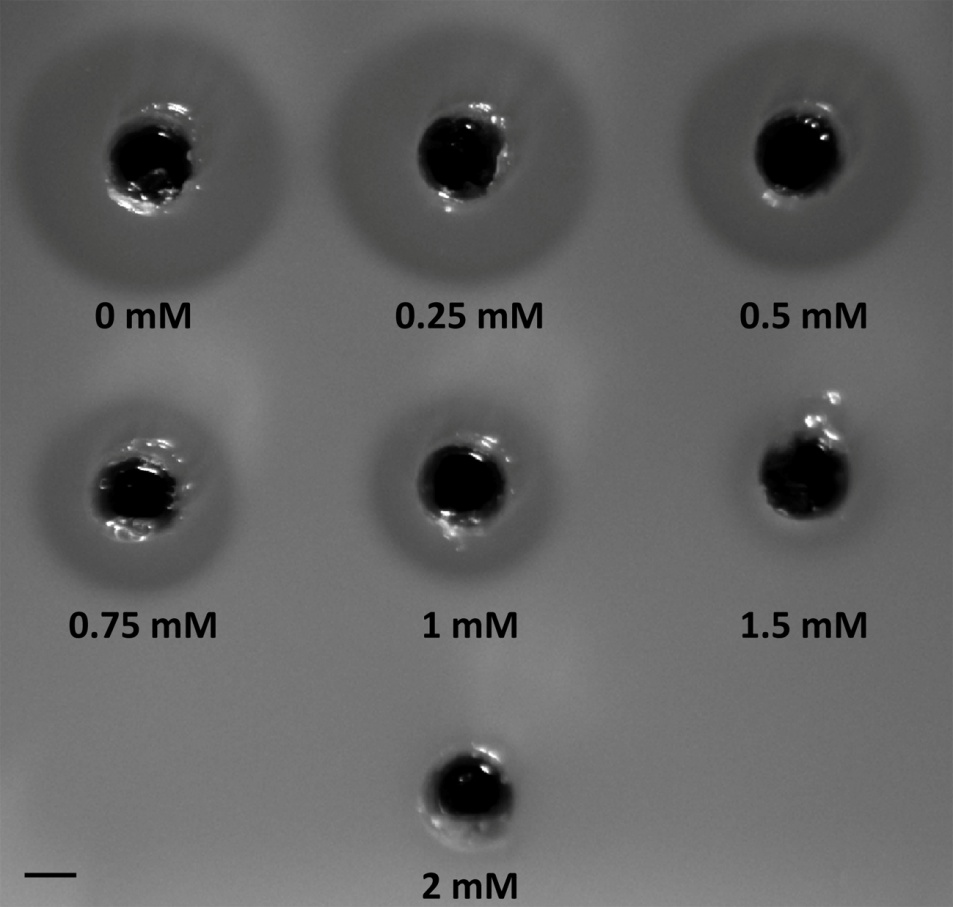


**Fig. S6) Indole-3-pyruvic acid inhibits andrimid biosynthesis in *Serratia plymuthica* A153.** Andrimid production by *S. plymuthica* A153 strain JH6 (zeamine negative) grown in minimal medium in the presence of increasing concentrations of IPA. For the assays, a *Bacillus subtilis* agar lawn was prepared and 400 µl of filter-sterilized supernatants were added to holes punched in the bioassay plates. Bar, 5 mm.


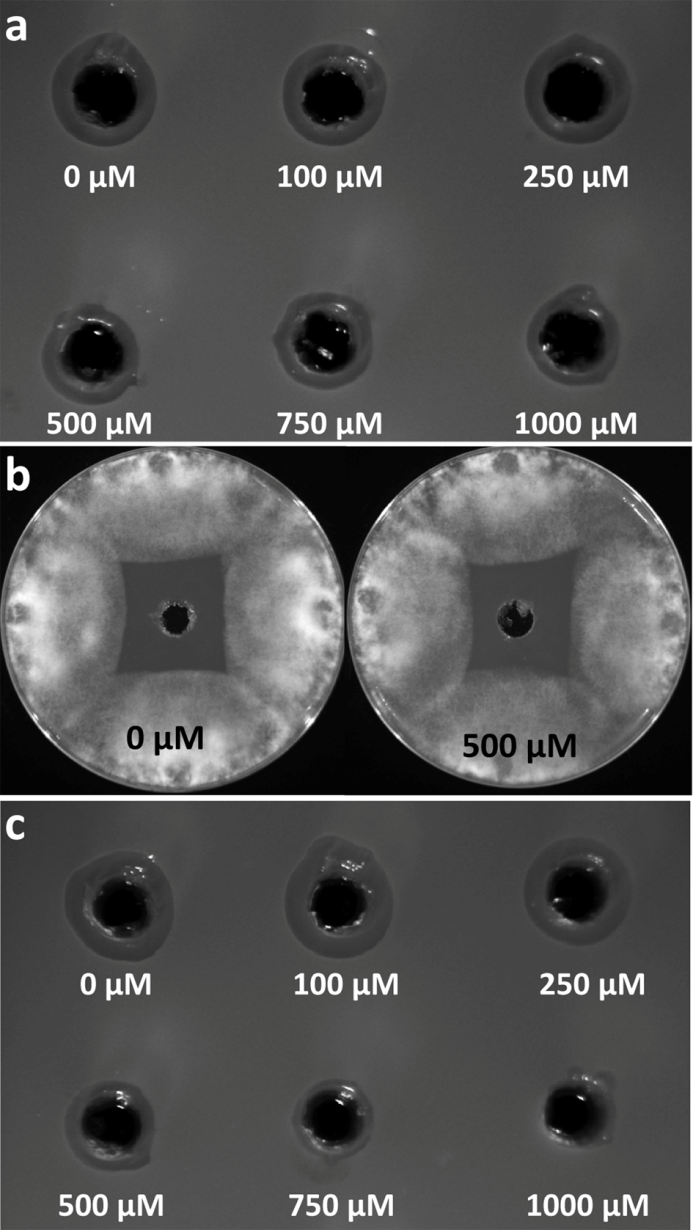


**Fig. S7) Influence of indole-3-acetic acid (IAA) and indole-3-pyruvic (IPA) acid on the biosynthesis of zeamine and oocydin A in *Serratia plymuthica* A153. a** & **c**, Bioassays of the production of zeamine by *S. plymuthica* A153 LVN2 (andrimid negative) in the presence of different concentrations of IAA (a) and IPA (c). **b**, Influence of IAA on the synthesis of the polyketide oocydin A. Bioactivities against *Bacillus subtilis* (a, c) and the oomycete *Pythium ultimum* (b) are shown. For the assays, 400 µl of filter-sterilized supernatants were added to holes punched into the bioassay plates.

**
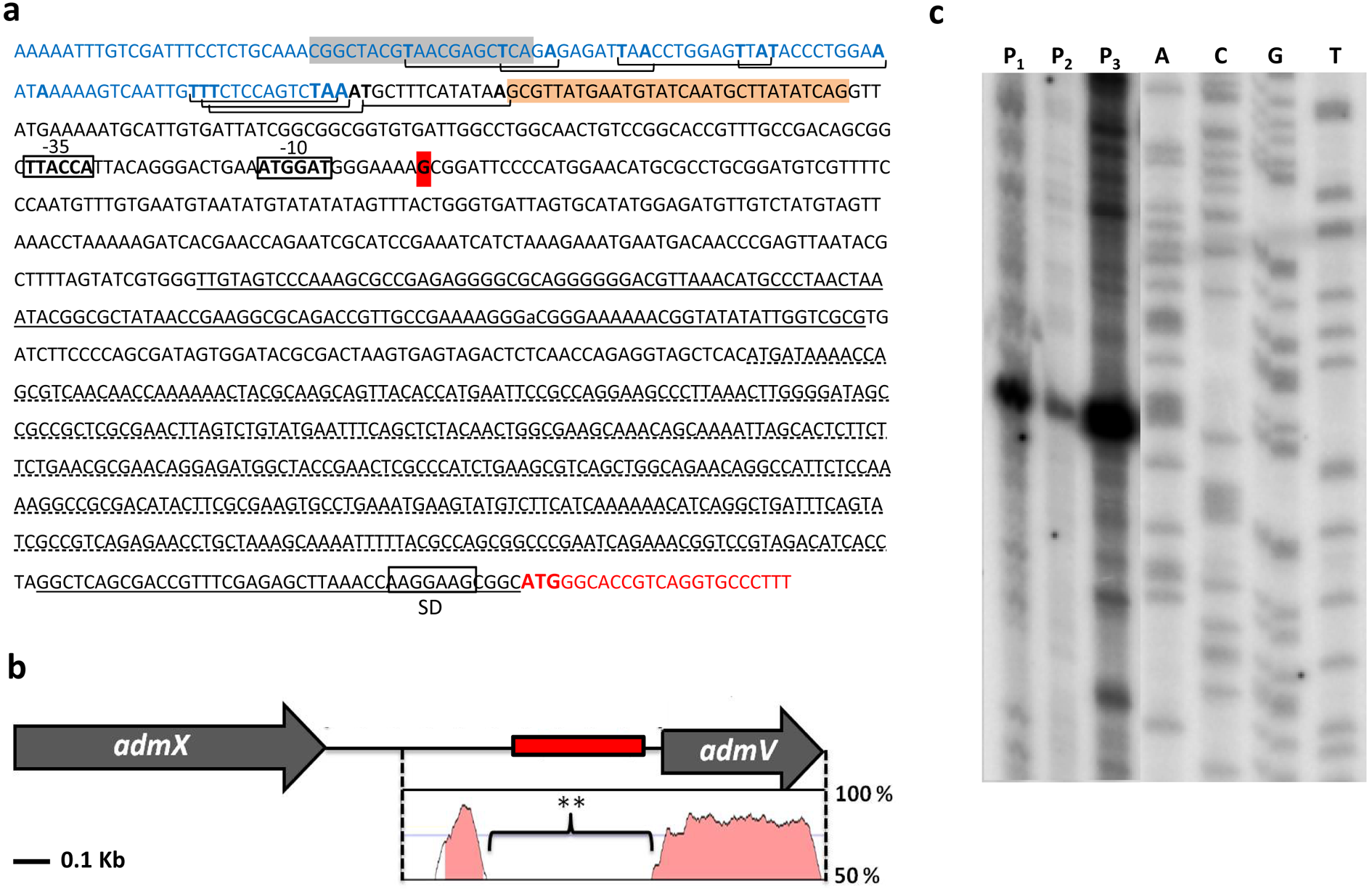
**

**Fig. S8) Characterization of the promoter region upstream the andrimid gene cluster of *Serratia plymuthica* A153. a**, Sequence of the *admX*-*admV* intergenic region harbouring the P*_adm_* promoter. Genes *admX* and *admV* appear in blue and red, respectively, with their respective stop (TAA, *admX*) and start (ATG, *admV*) codons bolded. The transcription start site of *admV* is highlighted in red. Putative -35 and -10 sequences are also indicated. DNA regions complementary to primers used to construct pMAMV269 (transcriptional fusion showing β-galactosidase activity) and pMAMV259 (fusion showing no transcriptional activity) are highlighted in grey and orange, respectively. The Shine-Dalgarno (AAGGAAG) sequence of *admV* is indicated with a box and the SD label. Sequences compatible with the consensus box of LTTR (T-N_11_-A)(3), which represent potential AdmX operator sites, are shown in bold and marked with brackets. Promoter regions showing homology to the *admV* promoter of the andrimid producing strain *Vibrionales* SWAT-3 (PATRIC Genome ID 391574.12) are underlined with continuous lines. Sequences showing homology to remnants of transposable genetic elements (i.e. IS3 transposases) are underlined with dashed lines. **b**, Schematic representation of *admX*, *admV* and the *admX*-*admV* intergenic region. DNA homology between the intergenic region *admX*-*admV* of *S. plymuthica* A153 and intergenic region *admX*-*admV* of *Vibrionales* SWAT-3 is also shown. Alignments were performed using wgVISTA(4). The red box indicates the location of sequences with homology to transposable genetic elements. **c**, Primer extension analysis using RNA from *Serratia plymuthica* A153 (P_1_), *Serratia plymuthica* Δ*admX* (P_2_) and *Serratia plymuthica* A153 (pMAMV282) (P_3_; plasmid containing the P*_adm_* promoter) in stationary phase of growth (OD_600_ = 4.0). A, C, G, T columns correspond to the sequencing ladder.

**
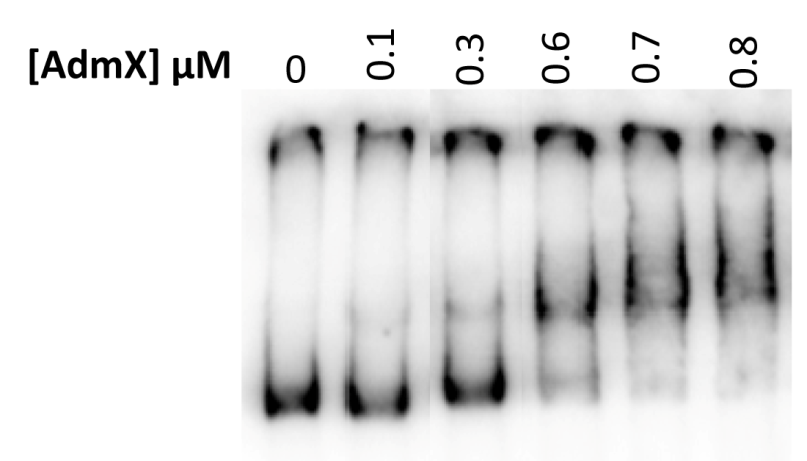
**

**Fig. S9) Binding of AdmX to P*_adm_* is not affected by the presence of indole-3-acetic acid.** Binding of AdmX to P*_adm_* in the presence of 1 mM indole-3-acetic acid. Observed binding behaviour was highly similar to that observed in the absence of IAA (Fig. 4). Size and location of the DNA fragment (DNA 1) used for this assays are provided in Fig. 4.


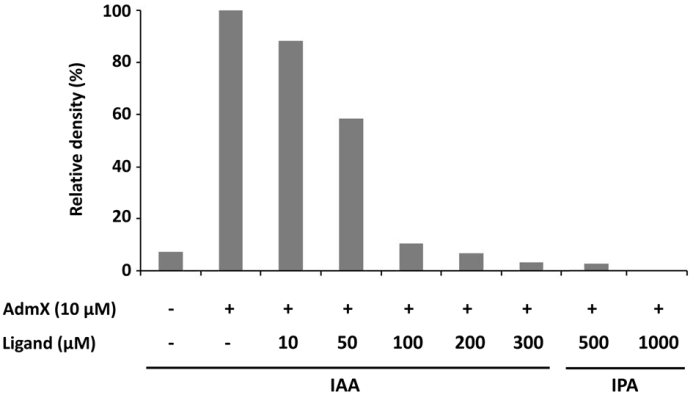


**Fig. S10) Densitometric analysis of *in vitro* transcription assays from P*_adm_* promoter shown in Fig. 5.** The quantification was done using the Quantity One Analysis software v.4.6.1 ([Bio-Rad Laboratories).](http://www.bio-rad.com/es-es/product/quantity-one-1-d-analysis-software?ID=1de9eb3a-1eb5-4edb-82d2-68b91bf360fb)


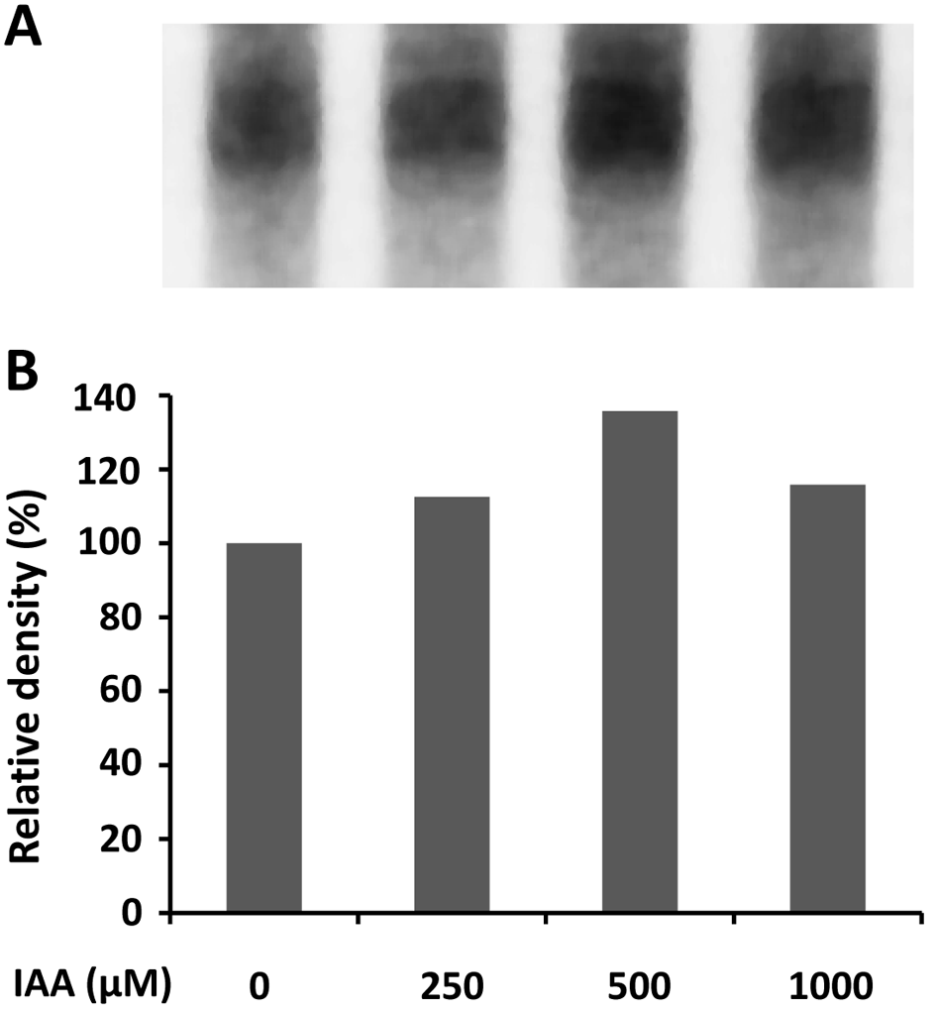


**Fig. S11) *In vitro* transcription assay of promoter P*_gap-1_* of *Pseudomonas aeruginosa* in the absence and presence of Indole-3-acetic acid (IAA). a**) Gel showing P*_gap-1_* transcripts. **b**) Densitometric analysis of above data. Experiments were conducted as reported in (5).


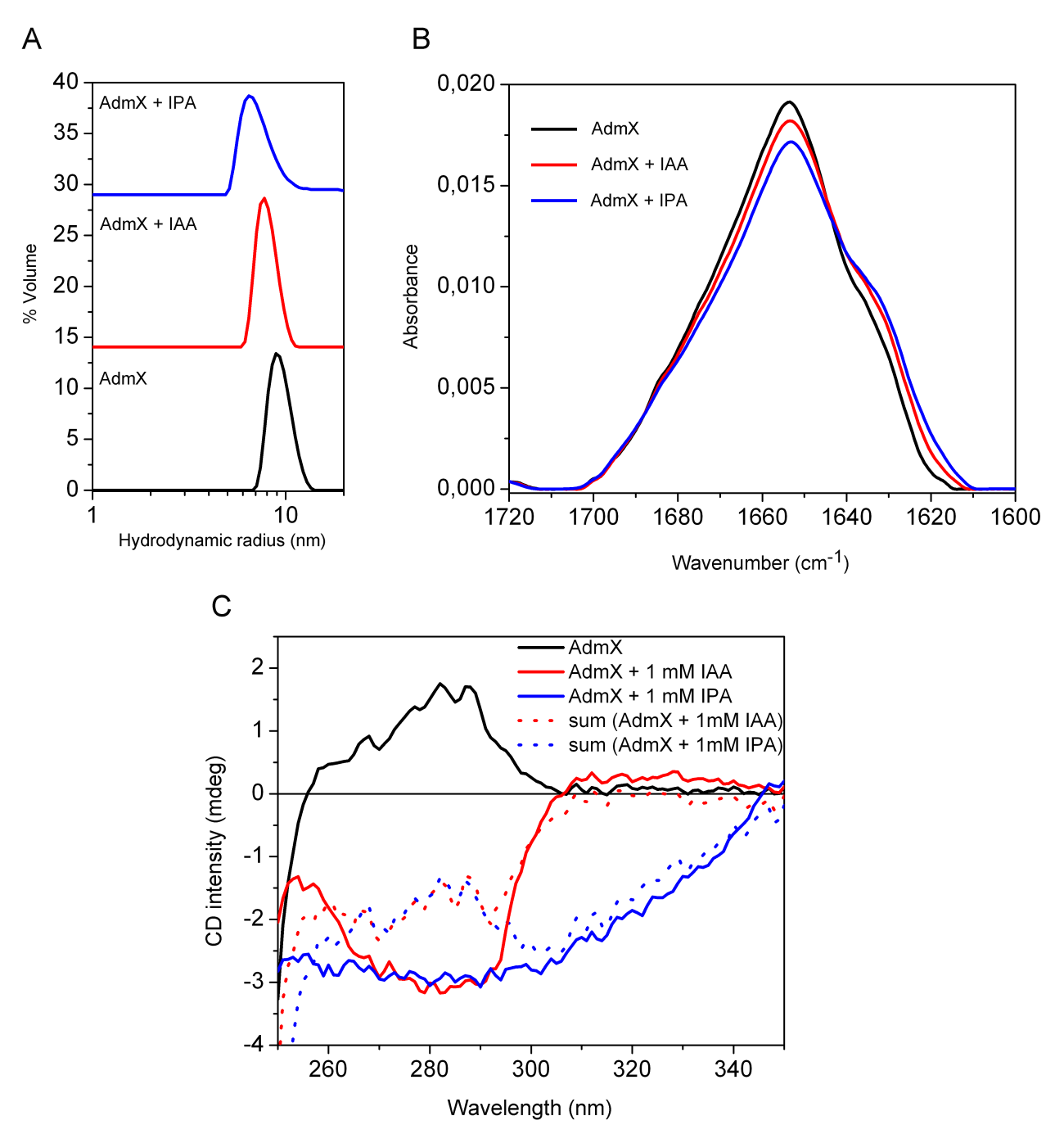


**Fig. S12) Effect of IAA and IPA binding on the particle size and secondary structure of AdmX.** **a**) Particle size distribution of AdmX (18.65 µM) in the absence and presence of IAA and IPA as determined by Dynamic Light Scattering. **b**) Attenuated Total Reflectance-Fourier Transform Infrared (ATR-FTIR) spectra of the amide I region of AdmX (29.22 µM) in the absence and presence of IAA and IPA. c) Near-UV circular dichroism spectra of AdmX (20 µM) in the presence and absence of IAA and IPA. The sums of the individual spectra are shown in dotted lines, whereas spectra of the protein ligand complexes are shown as continuous lines. In all experiments the final ligand concentration was of 1 mM.


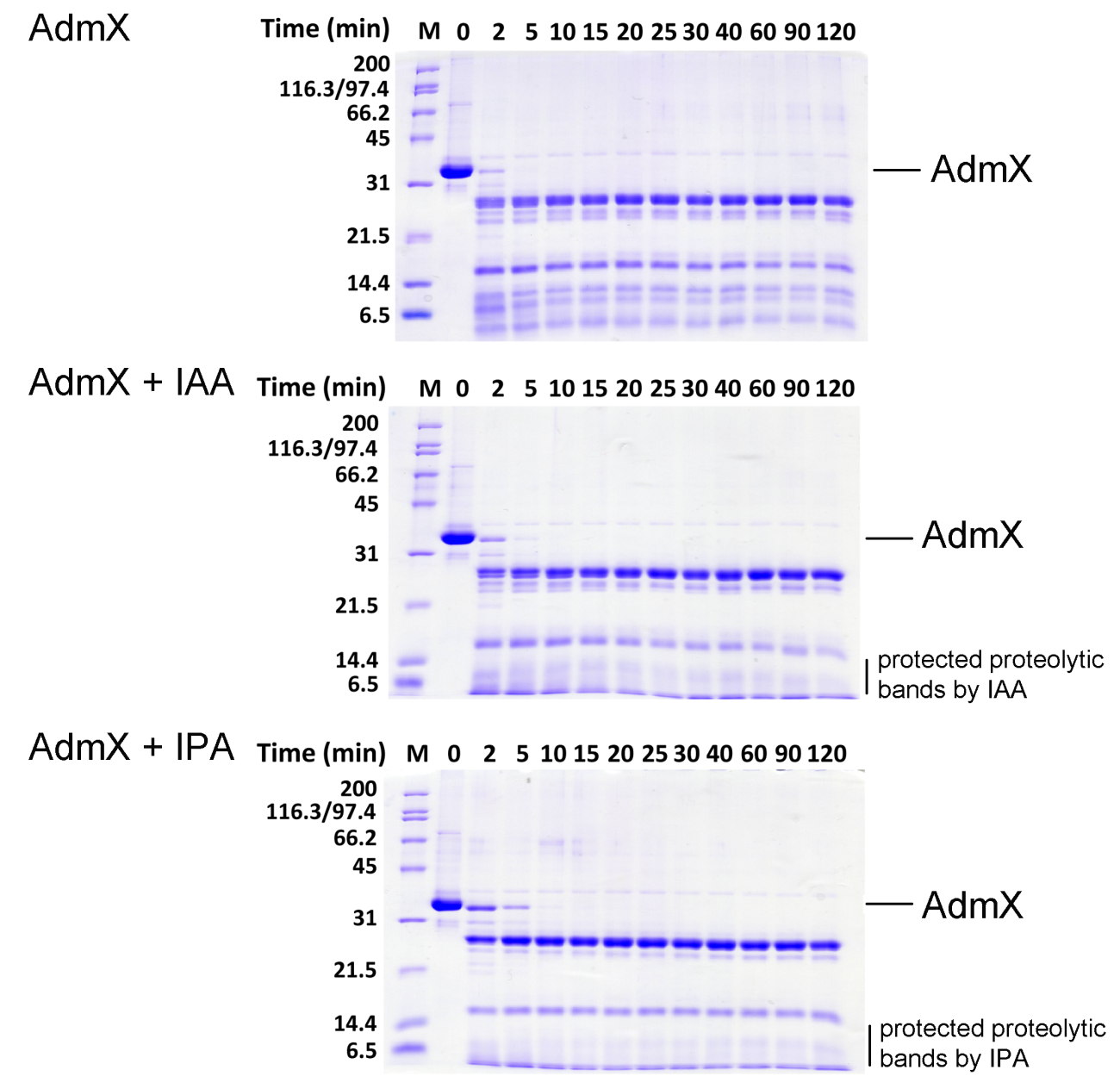


**Fig. S13) Limited proteolysis of AdmX in the absence and presence of IAA or IPA.** AdmX (10 µM) in the absence or presence of 1 mM IAA or IPA was incubated with a mixture of trypsin and α-chymotrypsin for 120 min. Samples were taken at regular intervals and analysed by electrophoresis on 15 % (w/v) SDS-PAGE gels.


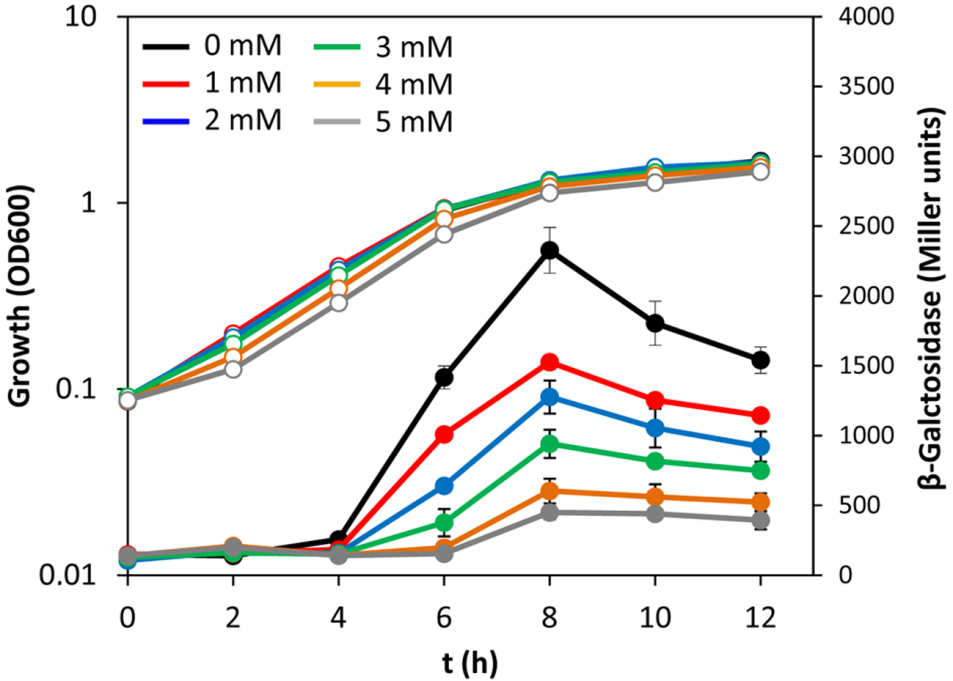


**Fig. S14) Indole-3-acetic acid regulates the expression of the andrimid biosynthetic cluster *in vivo*.** β-galactosidase activity (filled symbols) throughout growth measured from a chromosomal fusion *admK*::´*lacZ* in *Serratia plymuthica* A153 LacA in minimal medium with 15 mM glucose as carbon source at 25 ºC. Open symbols represent bacterial growth. Means and standard deviations are shown from of three biological replicates.

**
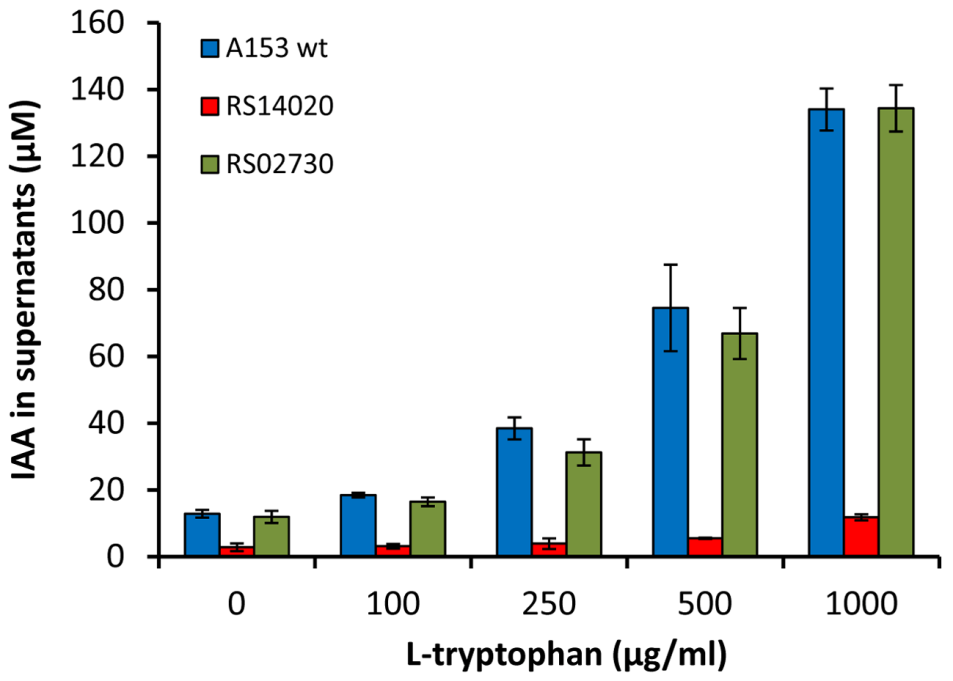
**

**Fig. S15) Indole-3-acetic acid (IAA) production by *Serratia plymuthica* A153 strains.** Strains RS14020 and RS02730 are in-frame deletion mutants defective in the genes encoding putative indole-3-pyruvate decarboxylases. Assays were performed in LB medium in the presence and absence of different concentrations of the IAA precursor L-tryptophan. Means and standard deviations of three biological replicates are shown. Samples were taken after 48 h incubations at 30 ºC.

**
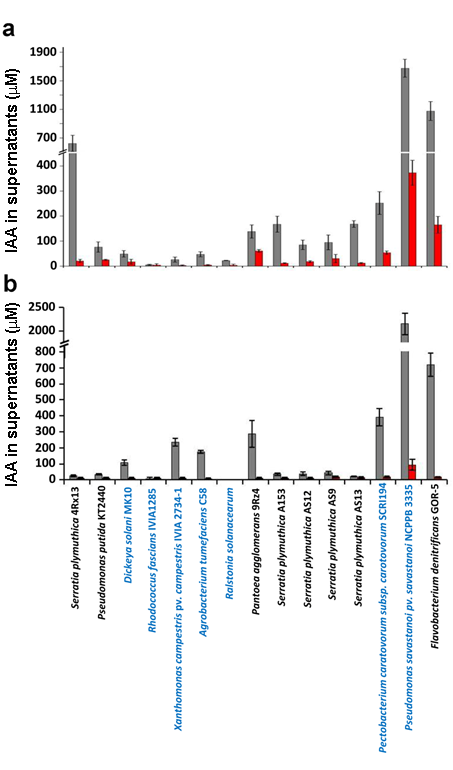
**

**Fig. S16) Indole-3-acetic acid production by different beneficial and pathogenic plant-associated bacterial strains.** Assays were performed in LB broth (**a**) and minimal media with 15 mM glucose as carbon source (**b**) in the presence (grey) and absence (red) of 1 mg/ml L-tryptophan. Bacterial phytopathogens are labelled in blue. Means and standard deviations of three biological replicates are shown. Samples were taken after 48 h incubations at 30 ºC.

**
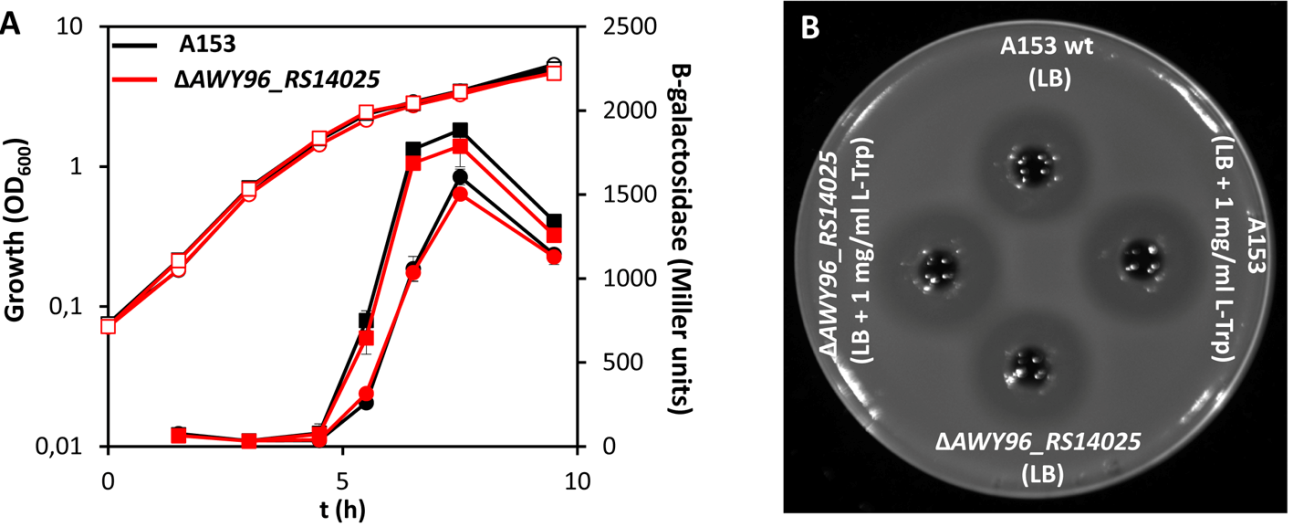
**

**Fig. S17) Influence of endogenous IAA synthesis on the production of andrimid in *Serratia plymuthica* A153. a**, β-galactosidase activity (filled symbols) throughout growth measured from a chromosomal fusion *admK*::´*lacZ* in *Serratia plymuthica* A153 LacA and its RS14020 derivative strain. The assays were done in LB medium at 25 ºC in the absence (circles) and presence (squares) of 1 mg/ml L-tryptophan. Open symbols represent bacterial growth. Data are the mean and standard deviation of three biological replicates. **b**, Influence of endogenous IAA on the andrimid-derived antibacterial properties against *Bacillus subtilis* in *S. plymuthica* A153 strains. For the assays, bacterial strains were grown in LB medium at 25 ºC in the presence or absence of 1 mg/ml of L-tryptophan. After 24 h of incubation, 400 µl of filter-sterilized supernatants were added to holes punched into the bioassay plates.


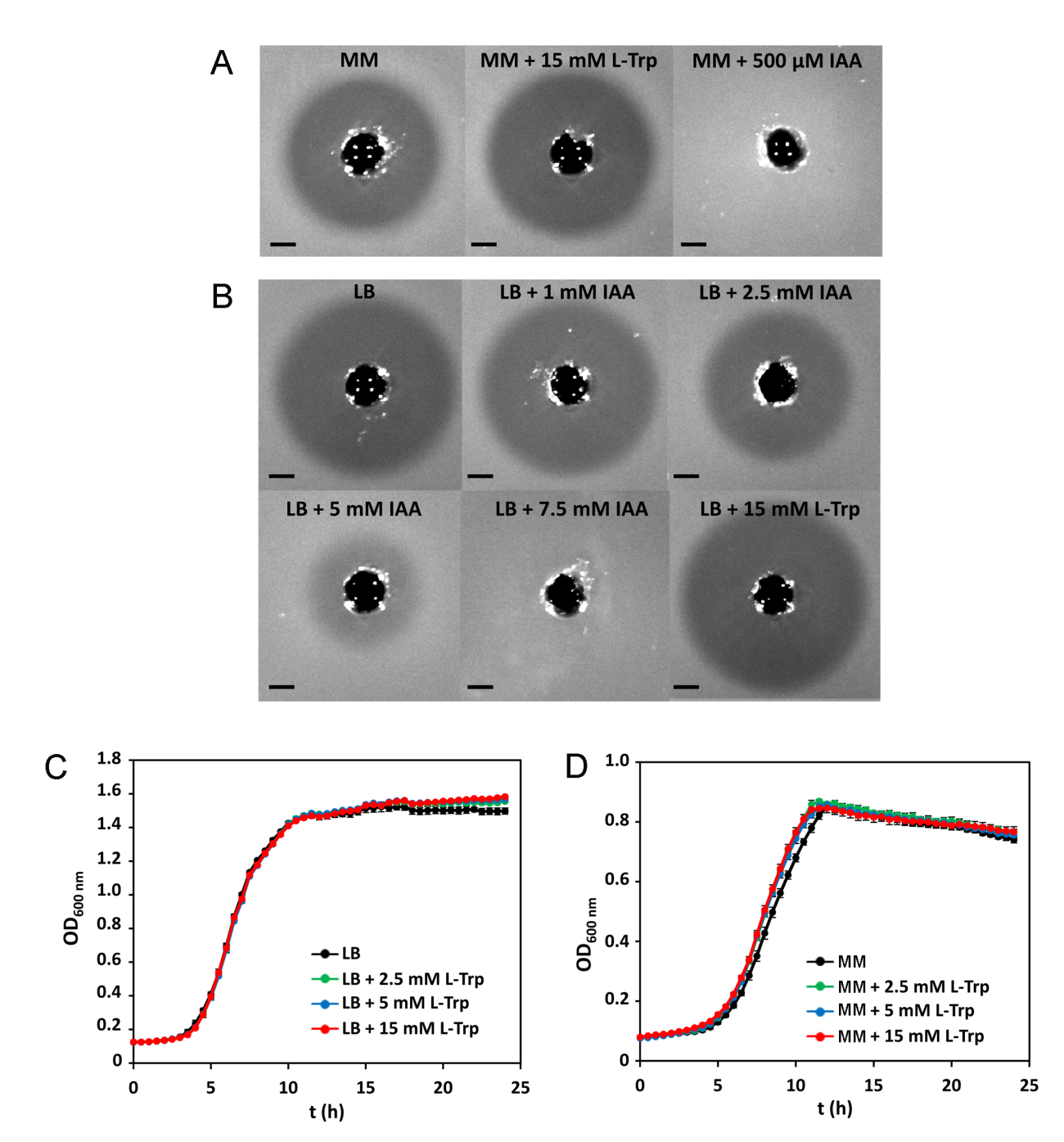


**Fig. S18)** **Andrimid production in different media in the presence and absence of indole-3-acetic acid and L-tryptophan.** Shown are bioassays for the production of andrimid by S*. plymuthica* A153 strain JH6 (zeamine negative) in minimal medium (MM) (**a**) and LB (**b**). For the assays, a *Bacillus subtilis* agar lawn was prepared and 400 μl of filter-sterilized supernatants were added to holes punched in the bioassay plates. Bars, 5 mm. Growth curves of *Serratia plymuthica* A153 in LB (**c**) and minimal medium (MM) supplemented with 10 mM glucose (**d**) in the absence and presence of different L-tryptophan concentrations. Data were recorded on a Bioscreen Microbiological Growth Analyser (Oy Growth Curves Ab Ltd, Helsinki, Finland) at 30 ºC. Shown are the mean and standard deviation of three biological replicates.


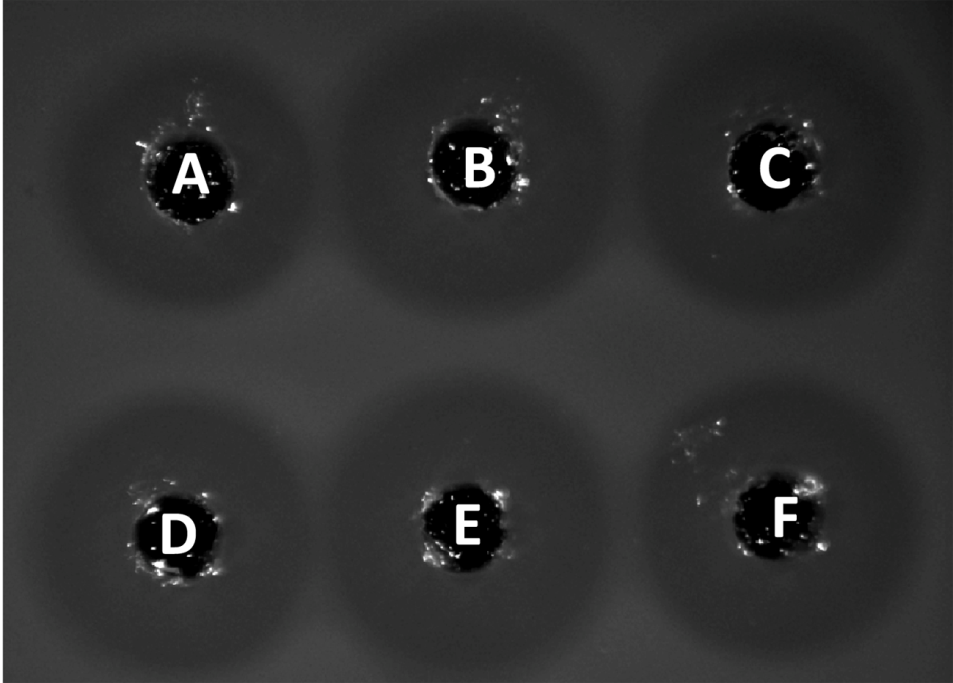


**Fig. S19) Influence of root extracts from *Arabidopsis thaliana* on the andrimid biosynthesis.** Andrimid production by *S. plymuthica* A153 strain JH6 (zeamine negative) grown in minimal medium in the absence (**A**) and presence of extracts from 166 mg of roots from *Arabidopsis thaliana* (**B**), *A. thaliana* *wei8/tar2* (reduced auxin production; **C**), *A. thaliana* *axr1* (reduced auxin production mutant; **D**), *A. thaliana* *sur2* (mutant with enhanced auxins levels; **E**) and IAA-exogenously treated *A. thaliana* (enhanced auxin levels; **F**). For the assays, a *Bacillus subtilis* agar lawn was prepared and 400 µl of filter-sterilized supernatants were added to holes punched in the bioassay plates.

*Pluralibacter_gergoviae*_(AIR01172) MKLRHLEIFYAVMTCGSLSRAAESLNISQPAASKALKSAEQKLGFPLFQRVRGKLLPSRE

*Serratia_plymuthica*_A153_(KYQ97099) MKLRHLEIFYTVMTCGSLSRAAESLNISQPAASKSLKNAELKLGFKLFQRVRGKLLPSRE

*Escherichia*_sp._B1147_(WP_064528456) MKLRHLEIFYAVMTCGTLSRAAESLNISQPAASKALKNAEQKLGFQLFQRVRGKLLPTSE

*Escherichia_fergusonii*_B253_(EGC07633) MKLRHLEIFYAVMTCGTLSRAAESLNISQPAASKALKNAEQKLGFQLFQRVRGKLLPTSE

*Klebsiella_pneumoniae*_BIDMC52_(EWD97089) MKLRHLEIFYAVMTCGSLSRAAEALNISQPAASKALKSAEMKLGFTLFQRVRGKLLPTSE

*Klebsiella_pneumoniae*_EGD-HP19-C_(EXF41166) MKLRHLEIFYAVMTCGSLSRAAEALNISQPAASKALKSAEMKLGFTLFQRVRGKLLPTSE

*Klebsiella_aerogenes*_KCTC2190_(YP_004593594) MKLRHLEIFYAVMTCGSLSRAAESLNISQPAASKALKNAELKLGFKLFQRVRGKLLPSSE

*Raoultella_ornithinolytica*_(AJF73714) MKLRHLEIFHAVMTCGTLSRAAESLNISQPAASKALKNAEMKLGFKLFQRVRGKLLPSSE

*Raoultella_terrigena*_(OMP90733) MKLRHLEIFHAVMTCGTLSRAAESLNISQPAASKALKNAEMKLGFKLFQRVRGKLLPSSE

*Raoultella_planticola*_(OAZ86055) MKLRHLEIFHAVMTCGTLSRAAESLNISQPAASKALKNAEQKLGFKLFQRVRGKLLPSSE

*********::*****:******:**********:**.** **** ***********: *

*Pluralibacter_gergoviae*_(AIR01172) ALTLYEKAQHIYQDLDSLRLLADNLARDPRARLSLGCLPSLGLSLVPGIVTDFYQQNANL

*Serratia_plymuthica*_A153_(KYQ97099) ALELFEKAQGIYQDLSNLRLLADNLARDPRAKFTLGCLPCLGLSLVPEIATDFYQQNSNL

*Escherichia*_sp._B1147_(WP_064528456) AITLFEKAQSIYHELDNLRLLADNLTRDPRARIALGCLPSLGLSLVPEIVTAFYQQNANL

*Escherichia_fergusonii*_B253_(EGC07633) ALTLFEKAQSIYHELDNLRLLADNLTRDPRARIALGCLPSLGLSLVPEIVTAFYQQNANL

*Klebsiella_pneumoniae*_BIDMC52_(EWD97089) AITLFEKAQSIYQDLDNLRLLADNLARDPRAKITLGCLPSLGLSLVPELVTDFYQQNSNL

*Klebsiella_pneumoniae*_EGD-HP19-C_(EXF41166) AITLFEKAQSIYQDLDNLRLLADNLARDPRAKITLGCLPSLGLSLVPELVTDFYQQNSNL

*Klebsiella_aerogenes*_KCTC2190_(YP_004593594) ALTLFEKAQSIYQDLDNLRLLADNLARDPRAKITLGCLPSLGLSLVPELVTDFYQQNSNL

*Raoultella_ornithinolytica*_(AJF73714) ALTLFEKAQNIYQDLDNLRLLADNLARDPRAKISLGCLPSLGLSLVPEMVTDFYQQNANL

*Raoultella_terrigena*_(OMP90733) ALTLFEKAQNIYQDLDNLRLLADNLARDPRAKISLGCLPSLGLSLVPEMVTDFYQQNANL

*Raoultella_planticola*_(OAZ86055) ALMLFEKAQNIYHDLDNLRLLADNLARDPRAKMSLGCLPSLGLSLVPELVTDFYQQNANL

*: *:**** **::*..********:*****:::*****.******* :.* *****:**

*Pluralibacter_gergoviae*_(AIR01172) VMTLSTEHTEMLIKKLALLEIDLALTLQPCEQGDILSHPIADVPLVYIDRDYRQGSVNIE

*Serratia_plymuthica*_A153_(KYQ97099) VMTLTAEHTETLVKKLDLREIDLALTMQPVQQGDIMATLIAEVPLVYVDKDYRQGAVEID

*Escherichia*_sp._B1147_(WP_064528456) VMTLTTEHTETLVKKLDLREIDLALTLQPIQQGEIMTTLIAEVPLVYIDRDYRQGAVEID

*Escherichia_fergusonii*_B253_(EGC07633) VMTLTTEHTETLVKKLDLREIDLALTLQPIQQGEIMTTLIAEVPLVYIDRDYRQGAVEID

*Klebsiella_pneumoniae*_BIDMC52_(EWD97089) VMTLTTEHTKTLVKKLDLREIDLALTLQPVQQGEILTTLIAEVPLVYIDRHYRQGAVEID

*Klebsiella_pneumoniae*_EGD-HP19-C_(EXF41166) VMTLTTEHTETLVKKLDLREIDLALTLQPVQQGEILTTLIAEVPLVYIDRHYRQGAVEID

*Klebsiella_aerogenes*_KCTC2190_(YP_004593594) VMTLTTEHTETLVKKLDLREIDLALTLQPVQQGEIITTLIAEVPLVYIDRDYRQGAVDIK

*Raoultella_ornithinolytica*_(AJF73714) VMTLTTEHTETLVKKLDLREIDLALTLQPVQQGEIITTTIAEVPLVYIDRDYRQGVVAID

*Raoultella_terrigena*_(OMP90733) VMTLTTEHTETLVKKLDLREIDLALTLQPVQQGEIITTTIAEVPLVYIDRDYRQGVVAID

*Raoultella_planticola*_(OAZ86055) VMTLTTEHTETLVKKLDLREIDLALTLQPVQQGEITSTAIAEVPLVYIDRDYRQGEVVIE

****::***: *:*** * *******:** :**:* : **:*****:*:.**** * *.

*Pluralibacter_gergoviae*_(AIR01172) EIDQRRWISPGNHSLSSAIAQHRHFSMTRLNVQTYYMATEFVKRGMGCSITDIFSARNNL

*Serratia_plymuthica*_A153_(KYQ97099) SIDQQRWISPGLDSLSTAIAAHRVFPATGLNVETCYMAMEFVKRGVGCCITDIFSARHSL

*Escherichia*_sp._B1147_(WP_064528456) KIDQQRWISPGPHSLSDAIAKRRDFLTTRLNVQTYYMATEFVKRGMGCSITDIFSARHNL

*Escherichia_fergusonii*_B253_(EGC07633) KIDQQRWISPGPHSLSDAIAKRRDFLTTRLNVQTYYMATEFVKRGMGCSITDIFSARHNL

*Klebsiella_pneumoniae*_BIDMC52_(EWD97089) QIDQQRWISPGPHSLSAAIATRRDFSTTRLNVQTYYMATEFVKRGMGCSITDIFSAQHNL

*Klebsiella_pneumoniae*_EGD-HP19-C_(EXF41166) QIDQQRWISPGPHSLSAAIATRRDFSTTRLNVQTYYMATEFVKRGMGCSITDIFSAQHNL

*Klebsiella_aerogenes*_KCTC2190_(YP_004593594) EIDQQRWISPGPHSLSAAIATRRDFSTTRLNVQTYYMATEFVKRGIGCSITDIFSARHNL

*Raoultella_ornithinolytica*_(AJF73714) EIDQQRWISPGPHSLSAAIATRRDFSTTRLNVQTYYMATEFVKRGMGCSITDIFSARHNL

*Raoultella_terrigena*_(OMP90733) EIDQQRWISPGPHSLSAAIATRRDFSTTRLNVQTYYMATEFVKRGMGCSITDIFSARHNL

*Raoultella_planticola*_(OAZ86055) EIDQQRWISPGPHSLSAAIATRRDFSTTRLNVQTYYMATEFVKRGMGCSITDIFSARHNL

.***:****** .*** *** :* * * ***:* *** ******:**.*******::.*

*Pluralibacter_gergoviae*_(AIR01172) PPQMIHPLTPAVKVTLCLLRRSDISLSPVAQKFVDFLCQTLRQQIAAINSELYPENKKSI

*Serratia_plymuthica*_A153_(KYQ97099) TPEMIHQISPPMKIDLYLLRRADASLSPVTQKFVDFLCKRLRNELREINLELYPGNKKSI

*Escherichia*_sp._B1147_(WP_064528456) PAETIHPIEPPMKIDLCLLRRADVSLSPITQKFVDFLCQQLRQQLRAINLELYPENKKSI

*Escherichia_fergusonii*_B253_(EGC07633) PAETIHPIEPPMKIDLCLLRRADVSLSPITQKFVDFLCQQLRLQLRAINLELYPENKKSI

*Klebsiella_pneumoniae*_BIDMC52_(EWD97089) APEMIHPITPPMAINLCLLRRADVSLSPMAQKFVDFLCQRLRQQLKEINLRLYPDHKKSI

*Klebsiella_pneumoniae*_EGD-HP19-C_(EXF41166) APEMIHPITPPMAINLCLLRRADVSLSPMAQKFVDFLCQRLRQQLKEINLRLYPGHKKSI

*Klebsiella_aerogenes*_KCTC2190_(YP_004593594) SPEMIHPITPPMKINLCLLRRADVSLSPITQKFVDFLCKQLRQQLKEINLQLYPDHKKSI

*Raoultella_ornithinolytica*_(AJF73714) TPTMIHPISPPMTIPLCLLRRADVSLSPIAHKFVDFICRQLRKQLQEINLGLYPENKKSI

*Raoultella_terrigena*_(OMP90733) TPTMIHPISPPMTIPLCLLRRADVSLSPIAHKFVDFICQQLRKQLQEINLGLYPENKKSI

*Raoultella_planticola*_(OAZ86055) APAMIHPIAPPMMITLCLLRRADVSLSPISQKFVDFLCIRLRQQLQEINLELYPEHKKSI

** : * : : * ****:* ****:::*****:* ** :: ** *** :****

*Pluralibacter_gergoviae*_(AIR01172) APCG---

*Serratia_plymuthica*_A153_(KYQ97099) VSPV---

*Escherichia*_sp._B1147_(WP_064528456) APQA---

*Escherichia_fergusonii*_B253_(EGC07633) APQA---

*Klebsiella_pneumoniae*_BIDMC52_(EWD97089) APLG---

*Klebsiella_pneumoniae*_EGD-HP19-C_(EXF41166) APLG---

*Klebsiella_aerogenes*_KCTC2190_(YP_004593594) APLG---

*Raoultella_ornithinolytica*_(AJF73714) APQG---

*Raoultella_terrigena*_(OMP90733) APQG---

*Raoultella_planticola*_(OAZ86055) APQRQML

.

**Fig. S20) Multiple sequence alignment of proteins homologous to AdmX.** Protein GenBank accession numbers are shown in parentheses. Sequences were aligned using Clustal Omega (6).

**Table S1) Bacteria, oomycete, fungi, plants and phages used in this study**

| **Bacteria/oomycete/plant/phage** | **Genotype or relevant characteristic^a^** | **Reference or source^a^** |
| --- | --- | --- |
| *Escherichia coli* DH5α | *supE44 lacU169*(*Ø80lacZΔ M15*) *hsdR17* (r_K_^−^m_K_^−^) *recA1* *endA1* *gyrA96 thi-1* *relA1* | (7) |
| *E. coli* CC118λ*pir* | *araD* Δ(*ara*, *leu*) Δ*lacZ74* *phoA20* *galK* *thi-1* *rspE* *rpoB* *argE* *recA1* λ*pir* | (8) |
| *E. coli* HH26 | Mobilizing strain for conjugal transfer | (9) |
| *E. coli* β2163 | F^-^ RP4-2-Tc::Mu Δ*dapA*::(*erm-pir*); Km^R^ Em^R^ | (10) |
| *E. coli* BL21-AI | F^-^ *ompT hsdS*_B_ (r_B_^-^ m_B_^-^) *gal dcm araB*::*T7RNAP-tetA* | Invitrogen |
| *Serratia plymuthica* A153 | Wild type; rhizosphere isolate | (11) |
| LacA | A153 Δ*lacZ* (1470 bp Δ) | (12) |
| LVN2 | A153 Δ*lacZ*, *admK*::Tn-KRCPN1*lacZ*; Km^R^ | (13) |
| A153JH6 | A153 Δ*lacZ*, *zmn13*::Tn-KRCPN1*lacZ*, zeamine negative; Km^R^ | (14) |
| ANDX | A153 Δ*admX* | (13) |
| RS02730 | A153 Δ*lacZ*, Δ*AWY96_RS02730* | This study |
| RS14020 | A153 Δ*lacZ* , Δ*AWY96_14020* | This study |
| RS14020VN2 | A153 Δ*lacZ*, Δ*AWY96_14020*, *admK*::Tn-KRCPN1*lacZ*; generated by transduction using ϕMAM1; Km^R^ | This study |
| *Agrobacterium tumefaciens* C58 | Wild-type; plant pathogen | E. Min Lai (Academia Sinica, Taiwan) |
| *Bacillus subtilis* JH642 | *pheA1 trpC2* | J.A. Hoch |
| *Dickeya solani* MK10 | Wild-type; plant pathogen | (15) |
| *Flavobacterium denitificans* GOR-5 | Wild-type; plant-associated | Bio-Iliberis R&D |
| *Pantoea agglomerans* 9Rz4 | Wild-type; rhizospheric bacterium | (16) |
| *Pectobacterium carotovorum subsp. carotovorum* SCRI194 | Wild-type; plant pathogen | M. Milagros-López (IVIA, Spain) |
| *Pseudomonas putida* KT2440 | Wild-type; prototroph | (17) |
| *Pseudomonas savastanoi* pv*. savastanoi* NCPPB3335 | Wild-type; plant pathogen | (18) |
| *Ralstonia solanacearum* | Wild-type; plant pathogen | M. Milagros-López (IVIA, Spain) |
| *Rhodococcus fascians* IVIA 1285 | Wild-type; plant pathogen | M. Milagros-López (IVIA, Spain) |
| *Serratia plymuthica* AS9 | Wild-type; rhizospheric bacterium | (19) |
| *Serratia plymuthica* AS12 | Wild-type; rhizospheric bacterium | (20) |
| *Serratia plymuthica* AS13 | Wild-type; rhizospheric bacterium | (21) |
| *Serratia plymuthica* 4Rx13 | Wild-type; rhizospheric bacterium | (16) |
| *Xanthomonas campestris* pv. *campestris* IVIA 2734-1 | Wild-type; plant pathogen | M. Milagros-López (IVIA, Spain) |
| **Oomycete strains** | | |
| *Pythium ultimum* | Wild-type; plant pathogen | C.A. Gilligan |
| **Plant lines** | | |
| *Arabidopsis thaliana* | Wild-type; parent line Col (Columbia) | Lab stock |
| *Arabidopsis thaliana* *wei8/tar2* | Reduced auxin production mutant | (22) |
| *Arabidopsis thaliana* *axr1-3* | Reduced auxin production mutant | (23) |
| *Arabidopsis thaliana* *sur2* | Mutant with enhanced auxins levels | (24) |
| **Phages** | | |
| ϕMAM1 | Generalized transducing phage for *S. plymuthica* A153 | (25) |

### *^a^*Km, kanamycin; Em, erythromycin.

**Table S2) Plasmids used in this study.**

| **Plasmid** | **Relevant characteristic^a^** | **Primers used for cloning** | **Source** |
| --- | --- | --- | --- |
| pKNG101 | Sm^R^; *oriR6K mob sacBR* |  | (9) |
| pUC18Not | Ap^R^; identical to pUC18 but with two NotI sites flanking pUC18 polylinker |  | (8) |
| pNJ5000 | Tc^R^; Mobilizing plasmid used in marker exchange |  | (26) |
| pMP220 | Tc^R^; *oriRK2 ′lacZ* |  | (27) |
| pET28b(+) | Km^R^; Protein expression plasmid |  | Novagen |
| pET29b(+) | Km^R^; Protein expression plasmid |  | Novagen |
| pTE103 | Ap^R^; Plasmid with a strong T7 terminator downstream of  the multiple cloning site. Used on *in vitro* transcription assays. |  | (28) |
| pMAMV232 | Km^R^; pET29b(+) derivative containing a DNA fragment encoding full length AdmX. C*-*terminal His6*-*tag. | 1,2 | This study |
| pMAMV235 | Km^R^; pET28b(+) derivative containing a DNA fragment encoding AdmX-LBD. N*-*terminal His6*-*tag. | 3,4 | This study |
| pMAMV237 | Tc^R^; a 447 bp region of the *admX-admV* intergenic region was cloned into the KpnI/SphI sites of pMP220 | 5,6 | This study |
| pMAMV259 | Tc^R^; a 979 bp region of the *admX-admV* intergenic region was cloned into the KpnI/SphI sites of pMP220 | 6,7 | This study |
| pMAMV269 | Tc^R^; a 1071 bp region containing the *adm* promoter was cloned into the KpnI/SphI sites of pMP220 | 6,8 | This study |
| pMAMV279 | Tc^R^; a 334 bp region containing the *adm* promoter was cloned into the KpnI/SphI sites of pMP220 | 8,9 | This study |
| pMAMV280 | Tc^R^; a 509 bp region containing the *adm* promoter was cloned into the KpnI/SphI sites of pMP220 | 8,10 | This study |
| pMAMV281 | Tc^R^; a 534 bp region containing the *adm* promoter was cloned into the KpnI/SphI sites of pMP220 | 9,11 | This study |
| pMAMV282 | Tc^R^; a 714 bp region containing the *adm* promoter was cloned into the KpnI/SphI sites of pMP220 | 10,11 | This study |
| pMAMV265 | Ap^R^; 1.4-kb PCR product containing a 1410 bp in frame deletion of *AWY96_RS02730* of A153 inserted into the EcoRI/HindIII sites of pUC18Not | 12,13 and 14,15 | This study |
| pMAMV266 | Ap^R^; 1.5-kb PCR product containing a 1365 bp in frame deletion of *AWY96_14020* of A153 inserted into the EcoRI/HindIII sites of pUC18Not | 16,17 and 18,19 | This study |
| pMAMV267 | Sm^R^; 1.6-kb NotI fragment of pMAMV265 was cloned at the same site in pKNG101 |  | This study |
| pMAMV268 | Sm^R^; 1.6-kb NotI fragment of pMAMV266 was cloned at the same site in pKNG101 |  | This study |
| pMAMV286 | Ap^R^; a 714 bp region containing the *adm* promoter was cloned into the EcoRI/PstI sites of pTE103 | 20,21 | This study |

*^a^*Ap, ampicillin; Km, kanamycin; Sm, streptomycin; Tc, tetracycline.

**Table S3) Oligonucleotides used in this study.**

| **No.** | **Name** | **Sequence (5´- 3')** | **Description/purpose** | **Source** |
| --- | --- | --- | --- | --- |
| 1 | AdmX-NdeI-F | TAATCATATGAAACTTCGCCATCTGG | Forward primer to clone *admX* into pET29b(+) | This study |
| 2 | AdmX-SalI-R | TAATGTCGACGACTGGAGAAACAATTGACTTTT | Reverse primer to clone *admX* into pET29b(+) | This study |
| 3 | AdmX-LBD-NdeI-F | TAATCATATGCAGGGTATCTATCAGGACCTG | Forward primer to clone the region encoding AdmX-LBD into pET28b(+) | This study |
| 4 | AdmX-LBD-BamHI-R | TAATGGATCCTCATCCAGGGTATAACTCCAGGTT | Reverse primer to clone the region encoding AdmX-LBD into pET28b(+) | This study |
| 5 | Adm(L)-KpnI-F | TAATGGTACCAGAGGTAGCTCACATGAT | Forward primer to clone a 447 bp region of the *adm* promoter into pMP220. Used in EMSA assays together primer No. 6. | This study |
| 6 | Adm-SphI-R | TAATGCATGCCGCTTCCTTGGTTT | Reverse primer to clone *adm* promoter regions into pMP220. Used in EMSA assays together primer No. 5. | This study |
| 7 | Adm(XL)-KpnI-F | TAATGGTACCGCGTTATGAATGTATCAATGCTTATATCAG | Forward primer to clone a 979 bp region of the *adm* promoter into pMP220. Used in EMSA assays together primer No. 10. | This study |
| 8 | Adm(XXL)-KpnI-F | TAATGGTACCCGGCTACGTAACGAGCTCAG | Forward primer to clone *adm* promoter regions into pMP220. Used in EMSA assays together primer No. 10. | This study |
| 9 | Adm-Prom-SphI-R | TAATGCATGCCTCCATATGCACTAATCACCCAGTAAAC | Reverse primer to clone *adm* promoter regions into pMP220 | This study |
| 10 | Adm-Prom2-SphI-R | TAATGCATGCGCGCCGTATTTAGTTAGGGCATG | Reverse primer to clone *adm* promoter regions into pMP220. Used in EMSA assays together primer No. 7 and 8. | This study |
| 11 | Adm-Prom-KpnI-F | TAATGGTACCCGTGGAGACCTGTTACATGGC | Forward primer to clone *adm* promoter regions into pMP220 | This study |
| 12 | IAA2173-EcoRI-F | TAATGAATTCATGCGTTGCAGGCTTTGATCC | Forward primer to clone upstream flanking region of *AWY96_RS02730* for in-frame deletion | This study |
| 13 | IAA2173-BamHI-R | TAATGGATCCGTGAGCAATCACGTGGTCGAG | Reverse primer to clone upstream flanking region of *AWY96_RS02730* for in-frame deletion | This study |
| 14 | IAA2173-BamHI-F | TAATGGATCCCAGGCATTGCGCGAAGTGG | Forward primer to clone downstream flanking region of *AWY96_RS02730* for in-frame deletion | This study |
| 15 | IAA2173-HindIII-R | TAATAAGCTTGCCTGGCGGACAAGACAGAAC | Reverse primer to clone downstream flanking region of *AWY96_RS02730* for in-frame deletion | This study |
| 16 | IAA3622-EcoRI-F | TAATGAATTCGCGTTGGCCGATCTTCGTC | Forward primer to clone upstream flanking region of *AWY96_14020* for in-frame deletion | This study |
| 17 | IAA3622-BamHI-R | TAATGGATCCCAACCGCGCATAACCGTCG | Reverse primer to clone upstream flanking region of *AWY96_14020* for in-frame deletion | This study |
| 18 | IAA3622-BamHI-F | TAATGGATCCCAGGATCGGCTGGCCTTTATCG | Forward primer to clone downstream flanking region of *AWY96_14020* for in-frame deletion | This study |
| 19 | IAA3622-HindIII-R | TAATAAGCTTCCATCAGCATCCGCAGGTTG | Reverse primer to clone downstream flanking region of *AWY96_14020* for in-frame deletion | This study |
| 20 | Adm-Prom-EcoRI-F | TAATGAATTCCGTGGAGACCTGTTACATGGC | Forward primer to clone promoter of *adm* into pTE103 | This study |
| 21 | Adm-Prom2-PstI-R | TAATCTGCAGGCGCCGTATTTAGTTAGGGCATG | Reverse primer to clone promoter of *adm* into pTE103 | This study |
| 22 | A2r | CTTTAGATGATTTCGGATGCGATTCTGG | Reverse primer for determination of transcriptional start point | This study |
| 23 | Edd/gap-F | CTGCAGTGCGGCGGGTGACT | Forward primer to amplify P_gap-1_ promoter region of Pseudomonas aeruginosa PAO1 and perform *in vitro* transcription assays | (5) |
| 24 | Edd/gap-R | AGATCTGTTGATGGCCAGGCGG | Reverse primer to amplify P_gap-1_ promoter region of Pseudomonas aeruginosa PAO1 and perform *in vitro* transcription assays | (5) |

**Table S4) Natural and synthetic auxins used in Differential Fluorimetry based ligand screening of AdmX-LBD.** The thermal shift induced by each compound is shown in Fig. 1.

| Number | Compound name | Abbreviation |
| --- | --- | --- |
| 1 | 1-ACETYL-5-BROMOINDOL-3-OL | 1Ac5BrI |
| 2 | 5-BROMOINDOLE-3-ACETIC ACID | 5BrIAA |
| 3 | 4-CHLOROINDOLE | 4ClI |
| 4 | 4-CHLOROINDOLE-3-ACETIC ACID | 4ClIAA |
| 5 | 5-CHLOROINDOLE-2-CARBOXYLIC ACID | 5ClI2CA |
| 6 | 5-CHLOROINDOLE-3-ACETIC ACID | 5ClIAA |
| 7 | 5-FLUOROINDOLE | 5FI |
| 8 | 5-FLUOROINDOLE-3-ACETIC ACID | 5FIAA |
| 9 | 5-FLUOROINDOLE-2-CARBOXYLIC ACID | 5FI2CA |
| 10 | 5-HYDROXYINDOLE-3-ACETIC ACID | 5OHIAA |
| 11 | 5-HYDROXYINDOLE-2-CARBOXYLIC ACID | 5OHI2CA |
| 12 | INDOLE-3-ACETIC ACID | IAA |
| 13 | INDOLE-3-ACETAMIDE | IAm |
| 14 | INDOLE-3-ACETIC ACID METHYL ESTER | IAAMe |
| 15 | INDOLE-3-ACETONE | IAc |
| 16 | INDOLE-3-ACETONITRILE | IAN |
| 17 | INDOLE-3-ACETYL-L-ALANINE | IAAla |
| 18 | INDOLE-3-ACETYL-L-GLUTAMIC ACID | IAGlu |
| 19 | INDOLE-3-ACETYL-L-ISOLEUCINE | IAIleu |
| 20 | INDOLE-3-ACETYL-L-LEUCINE | IALeu |
| 21 | INDOLE-3-ACETYL-L-PHENYLALANINE | IAPhe |
| 22 | INDOLE-3-ACETYL-L-PHENYLALANINE METHYL ESTER | IAPheMe |
| 23 | INDOLE-3-ACETYL-L-TRYPTOPHAN | IATrp |
| 24 | INDOLE-3-ACETYL-L-VALINE | IAVal |
| 25 | INDOLE-3-BUTYRIC ACID | IBA |
| 26 | INDOLE-3-BUTYRIC ACID POTASSIUM SALT | IBA-K |
| 27 | INDOLE-3-BUTYRIC ACID METHYL ESTER | IBAMe |
| 28 | INDOLE-3-CARBOXALDEHYDE | IAld |
| 29 | INDOLE-3-CARBINOL | IM |
| 30 | INDOLE-3-CARBOXYLIC ACID | I3CA |
| 31 | INDOLE-3-CARBOXYLIC ACID METHYL ESTER | I3CAMe |
| 32 | INDOLE-5-CARBOXYLIC ACID | I5CA |
| 33 | INDOLE-3-GLYOXYLIC ACID | IGA |
| 34 | INDOLE-3-PROPIONAMIDE | IPAM |
| 35 | INDOLE-3-PYRUVIC ACID | IPA |
| 36 | 3-INDOXYL-β-D-GLUCOPYRANOSIDE TRIHYDRATE | IOxGlc |
| 37 | MELATONIN | M |
| 38 | 5-METHOXYINDOLE-3-ACETIC ACID | 5MeOIAA |
| 39 | 4-CHLOROPHENOXYACETIC ACID | 4-CPA |
| 40 | 2,4,6-TRICHLOROBENZOIC ACID | 2,4,6-TB |
| 41 | 2-(2,4-DICHLOROPHENOXY)PROPIONIC ACID | 2,4-DP |
| 42 | 2-(4-CHLOROPHENOXY)ISOBUTYRIC ACID | PCIB |
| 43 | 4´,5,7-TRIHYDROXYFLAVONE | APIGENIN |

**Table S5) Secondary structural contents of AdmX in the presence and absence of IAA and IPA.** Data were obtained by the deconvolution of the amide I region of ATR-FTIR spectra shown in Fig. S12b.

| Protein | Secondary structure content (%) | | |
| --- | --- | --- | --- |
|  | α-helix | β-sheet | other^*^ |
| AdmX | 52.2 | 17.6 | 30.2 |
| AdmX + 1 mM IPA | 47.2 | 26.0 | 26.8 |
| AdmX + 1 mM IAA | 47.8 | 20.2 | 32.0 |

^*^other: β-turn, loop and random coil

**REFERENCES**

1. Finn, R.D., Coggill, P., Eberhardt, R.Y., Eddy, S.R., Mistry, J., Mitchell, A.L., Potter, S.C., Punta, M., Qureshi, M., Sangrador-Vegas, A., Salazar, G.A., Tate, J. and Bateman, A. (2016) The Pfam protein families database: towards a more sustainable future. *Nucleic Acids Res.,* **44**, D279-285.

2. Spaepen, S. and Vanderleyden, J. (2011) Auxin and plant-microbe interactions. *Cold Spring Harb. Perspect. Biol.,* **3**.

3. Maddocks, S.E. and Oyston, P.C. (2008) Structure and function of the LysR-type transcriptional regulator (LTTR) family proteins. *Microbiology.* **154**, 3609-3623.

4. Frazer, K.A., Pachter, L., Poliakov, A., Rubin, E.M. and Dubchak, I. (2004) VISTA: computational tools for comparative genomics. *Nucleic Acids Res.,* **32**, W273-279.

5. Daddaoua, A., Molina-Santiago, C., de la Torre, J., Krell, T. and Ramos, J.L. (2014) GtrS and GltR form a two-component system: the central role of 2-ketogluconate in the expression of exotoxin A and glucose catabolic enzymes in *Pseudomonas aeruginosa.* *Nucleic Acids Res.,* **42**, 7654-7663.

6. Sievers, F., Wilm, A., Dineen, D., Gibson, T.J., Karplus, K., Li, W., Lopez, R., McWilliam, H., Remmert, M., Soding, J., Thompson, J.D. and Higgins, D.G. (2011) Fast, scalable generation of high-quality protein multiple sequence alignments using Clustal Omega. *Mol. Syst. Biol.,* **7**, 539.

7. Woodcock, D.M., Crowther, P.J., Doherty, J., Jefferson, S., DeCruz, E., Noyer-Weidner, M., Smith, S.S., Michael, M.Z. and Graham, M.W. (1989) Quantitative evaluation of *Escherichia coli* host strains for tolerance to cytosine methylation in plasmid and phage recombinants. *Nucleic Acids Res.,* **17**, 3469-3478.

8. Herrero, M., de Lorenzo, V. and Timmis, K.N. (1990) Transposon vectors containing non-antibiotic resistance selection markers for cloning and stable chromosomal insertion of foreign genes in gram-negative bacteria. *J. Bacteriol.,* **172**, 6557-6567.

9. Kaniga, K., Delor, I. and Cornelis, G.R. (1991) A wide-host-range suicide vector for improving reverse genetics in gram-negative bacteria: inactivation of the *blaA* gene of *Yersinia enterocolitica*. *Gene,* **109**, 137-141.

10. Demarre, G., Guerout, A.M., Matsumoto-Mashimo, C., Rowe-Magnus, D.A., Marliere, P. and Mazel, D. (2005) A new family of mobilizable suicide plasmids based on broad host range R388 plasmid (IncW) and RP4 plasmid (IncPalpha) conjugative machineries and their cognate *Escherichia coli* host strains. *Res. Microbiol.,* **156**, 245-255.

11. Hökeberg, M., Gerhardson, B. and Johnsson, L. (1997) Biological control of cereal seed-borne diseases by seed bacterization with greenhouse-selected bacteria. *Eur. J. Plant Pathol.,* **103**, 25–33.

12. Matilla, M.A., Leeper, F.J. and Salmond, G.P. (2015) Biosynthesis of the antifungal haterumalide, oocydin A, in *Serratia*, and its regulation by quorum sensing, RpoS and Hfq. *Environ. Microbiol.,* **17**, 2993-3008.

13. Matilla, M.A., Nogellova, V., Morel, B., Krell, T. and Salmond, G.P. (2016) Biosynthesis of the acetyl-CoA carboxylase-inhibiting antibiotic, andrimid in *Serratia* is regulated by Hfq and the LysR-type transcriptional regulator, AdmX. *Environ. Microbiol.,* **18**, 3635-3650.

14. Hellberg, J.E., Matilla, M.A. and Salmond, G.P. (2015) The broad-spectrum antibiotic, zeamine, kills the nematode worm *Caenorhabditis elegans*. *Front. Microbiol.,* **6**, 137.

15. Pritchard, L., Humphris, S., Baeyen, S., Maes, M., Van Vaerenbergh, J., Elphinstone, J., Saddler, G. and Toth, I. (2013) Draft Genome Sequences of Four *Dickeya dianthicola* and Four *Dickeya solani* Strains. *Genome Announc.,* **1**.

16. Berg, G., Roskot, N., Steidle, A., Eberl, L., Zock, A. and Smalla, K. (2002) Plant-dependent genotypic and phenotypic diversity of antagonistic rhizobacteria isolated from different *Verticillium* host plants. *Appl. Environ. Microbiol.* **68**, 3328-3338.

17. Nakazawa, T. (2002) Travels of a *Pseudomonas*, from Japan around the world. *Environ. Microbiol.,* **4**, 782-786.

18. Rodriguez-Palenzuela, P., Matas, I.M., Murillo, J., Lopez-Solanilla, E., Bardaji, L., Perez-Martinez, I., Rodriguez-Moskera, M.E., Penyalver, R., Lopez, M.M., Quesada, J.M., Biehl, B.S., Perna, N.T., Glasner, J.D., Cabot, E.L., Neeno-Eckwall, E. and Ramos, C. (2010) Annotation and overview of the *Pseudomonas savastanoi* pv. *savastanoi* NCPPB 3335 draft genome reveals the virulence gene complement of a tumour-inducing pathogen of woody hosts. *Environ. Microbiol.,* **12**, 1604-1620.

19. Neupane, S., Hogberg, N., Alstrom, S., Lucas, S., Han, J., Lapidus, A., Cheng, J.F., Bruce, D., Goodwin, L., Pitluck, S., Peters, L., Ovchinnikova, G., Lu, M., Han, C., Detter, J.C., Tapia, R., Fiebig, A., Land, M., Hauser, L., Kyrpides, N.C., Ivanova, N., Pagani, I., Klenk, H.P., Woyke, T. and Finlay, R.D. (2012) Complete genome sequence of the rapeseed plant-growth promoting *Serratia plymuthica* strain AS9. *Stand. Genomic Sci.,* **6**, 54-62.

20. Neupane, S., Finlay, R.D., Alstrom, S., Goodwin, L., Kyrpides, N.C., Lucas, S., Lapidus, A., Bruce, D., Pitluck, S., Peters, L., Ovchinnikova, G., Chertkov, O., Han, J., Han, C., Tapia, R., Detter, J.C., Land, M., Hauser, L., Cheng, J.F., Ivanova, N., Pagani, I., Klenk, H.P., Woyke, T. and Hogberg, N. (2012) Complete genome sequence of *Serratia plymuthica* strain AS12. *Stand. Genomic Sci.,* **6**, 165-173.

21. Neupane, S., Finlay, R.D., Kyrpides, N.C., Goodwin, L., Alstrom, S., Lucas, S., Land, M., Han, J., Lapidus, A., Cheng, J.F., Bruce, D., Pitluck, S., Peters, L., Ovchinnikova, G., Held, B., Han, C., Detter, J.C., Tapia, R., Hauser, L., Ivanova, N., Pagani, I., Woyke, T., Klenk, H.P. and Hogberg, N. (2012) Complete genome sequence of the plant-associated *Serratia plymuthica* strain AS13. *Stand. Genomic Sci.,* **7**, 22-30.

22. Stepanova, A.N., Yun, J., Robles, L.M., Novak, O., He, W., Guo, H., Ljung, K. and Alonso, J.M. (2011) The Arabidopsis YUCCA1 flavin monooxygenase functions in the indole-3-pyruvic acid branch of auxin biosynthesis. *Plant Cell,* **23**, 3961-3973.

23. Lincoln, C., Britton, J.H. and Estelle, M. (1990) Growth and development of the *axr1* mutants of *Arabidopsis*. *Plant Cell* **2**, 1071-1080.

24. Barlier, I., Kowalczyk, M., Marchant, A., Ljung, K., Bhalerao, R., Bennett, M., Sandberg, G. and Bellini, C. (2000) The SUR2 gene of *Arabidopsis thaliana* encodes the cytochrome P450 CYP83B1, a modulator of auxin homeostasis. *Proc. Natl. Acad. Sci. U S A.,* **97**, 14819-14824.

25. Matilla, M.A. and Salmond, G.P. (2014) Bacteriophage phiMAM1, a viunalikevirus, is a broad-host-range, high-efficiency generalized transducer that infects environmental and clinical isolates of the enterobacterial genera *Serratia* and *Kluyvera*. *Appl. Environ. Microbiol.,* **80**, 6446-6457.

26. Grinter, N.J. (1983) A broad-host-range cloning vector transposable to various replicons. *Gene,* **21**, 133-143.

27. Spaink, H.P., Okker, R.J., Wijffelman, C.A., Pees, E. and Lugtenberg, B.J. (1987) Promoters in the nodulation region of the *Rhizobium leguminosarum Sym* plasmid pRL1JI. *Plant Mol. Biol.,* **9**, 27-39.

28. Elliott, T. and Geiduschek, E.P. (1984) Defining a bacteriophage T4 late promoter: absence of a "-35" region. *Cell,* **36**, 211-219.
